# Quantifying the Impact of Human Immunodeficiency Virus-1 Escape From Cytotoxic T-Lymphocytes

**DOI:** 10.1371/journal.pcbi.1000981

**Published:** 2010-11-04

**Authors:** Ulrich D. Kadolsky, Becca Asquith

**Affiliations:** Department of Immunology, Division of Medicine, Imperial College London, London, United Kingdom; Utrecht University, The Netherlands

## Abstract

HIV-1 escape from the cytotoxic T-lymphocyte (CTL) response leads to a weakening of viral control and is likely to be detrimental to the patient. To date, the impact of escape on viral load and CD4^+^ T cell count has not been quantified, primarily because of sparse longitudinal data and the difficulty of separating cause and effect in cross-sectional studies. We use two independent methods to quantify the impact of HIV-1 escape from CTLs in chronic infection: mathematical modelling of escape and statistical analysis of a cross-sectional cohort. Mathematical modelling revealed a modest increase in log viral load of 0.051 copies ml^−1^ per escape event. Analysis of the cross-sectional cohort revealed a significant positive association between viral load and the number of “escape events”, after correcting for length of infection and rate of replication. We estimate that a single CTL escape event leads to a viral load increase of 0.11 log copies ml^−1^ (95% confidence interval: 0.040–0.18), consistent with the predictions from the mathematical modelling. Overall, the number of escape events could only account for approximately 6% of the viral load variation in the cohort. Our findings indicate that although the loss of the CTL response for a single epitope results in a highly statistically significant increase in viral load, the biological impact is modest. We suggest that this small increase in viral load is explained by the small growth advantage of the variant relative to the wildtype virus. Escape from CTLs had a measurable, but unexpectedly low, impact on viral load in chronic infection.

## Author Summary

HIV, like many viruses, has evolved multiple strategies to evade immune surveillance. One of these strategies is the evolution of escape mutations which reduce the ability of the immune response to kill HIV-infected cells. But does HIV escape matter? Some believe that the accumulation of escape mutations leads to AIDS; many more believe escape is likely to be highly detrimental to human health. Yet, to date, it has not been possible to measure the impact of escape. We developed two independent methods to quantify the impact of escape on HIV viral load. Both methods showed that escape does lead to a detectable increase in viral load, but is unlikely to have a major impact on patient health as the increase is small. Indeed, only 6% of between-individual variation in viral load could be attributed to HIV escape. This work suggests that the current research focus on escape in chronic HIV infection might be out of proportion to its importance with other factors playing a more significant role in determining viral load.

## Introduction

The CTL response is thought to play a role in firstly reducing [1], [2] and then controlling the level of HIV-1 viraemia. Evidence for the protective role of CTLs in humans includes HLA class I heterozygous advantage [3], the evolution of multiple viral mechanisms to evade CTL surveillance [4]–[6] and the observation of statistically significant associations between possession of certain HLA class I alleles and the rate of disease progression [7] although the size of this CTL-mediated protective effect is contentious [8]–[10]. The pressure exerted by CTLs on HIV-1 leads to frequent [11], [12] selection of HLA class I-associated viral polymorphisms that result in reduced or abolished CTL recognition, a phenomenon known as HIV-1 “escape” from the CTL response [11], [13]–[17]. It seems likely that HIV escape would be detrimental for the host, yet the impact of escape on viral load or CD4^+^ T-cell count is unclear.

Longitudinal studies following viral escape in HIV-1 infected individuals have shown no conclusive results [18]–[21], largely because of data scarcity. Cross-sectional studies show a relationship between HLA-associated polymorphisms in *pol* and high viral load [11], and between HLA-associated polymorphisms in *gag* and low CD4^+^ count [22] and high viral load [23]. However, as acknowledged by the authors themselves [23], it is impossible to interpret these results because the direction of causality is unknown. That is to say, it is unclear whether the negative correlation between the number of viral polymorphisms and CD4^+^ T-cell count arises because a high number of polymorphisms leads to poor CTL control and greater immunosuppression, or simply because these individuals have been infected for a longer time, resulting in a lower CD4^+^ T-cell count and, simultaneously, a greater opportunity to develop mutations. Similarly, the association between high viral load and HLA-associated polymorphisms may be due to viral strains escaping the immune response which results in an increase in viral load, or it may be that high viral load and high viral replication allows for more mutations to accumulate. Furthermore, recent work demonstrated a correlation between rate of progression to AIDS and the replication rate of the infecting strain [24], [25]. This introduces another potential confounding factor into associations between HLA-associated polymorphisms and surrogate markers of disease progression. Therefore, in order to interpret the relationships between HLA-associated polymorphisms and viral load and/or CD4+ count, it is essential to adjust for the number of mutation events that have occurred in individuals.

Hence, because of limited longitudinal data and difficulties in interpreting cross-sectional data, the consequences of HIV-1 escape from the CTL response are still unclear. The aim of this work was to quantify the impact of HIV escape on viral load in chronic infection.

## Results

### Predicted increase in viral load upon escape is modest

We used an extension of the Perelson/De Boer model of HIV-1 dynamics [26] to simulate the outgrowth of an HIV-1 variant which has escaped a single CTL response. This enabled us, firstly, to check our intuition that escape must lead to an increase in viral load; secondly, to obtain an order of magnitude estimate of the predicted increase in viral load upon escape; and thirdly, to test whether a small increase in viral load upon escape could be explained solely by the outgrowth rate of the variant, or whether it was necessary to postulate additional mechanisms (e.g. the generation of *de novo* CTL responses which recognise the variant and subsequently reduce viral load).

**Figure 1:**
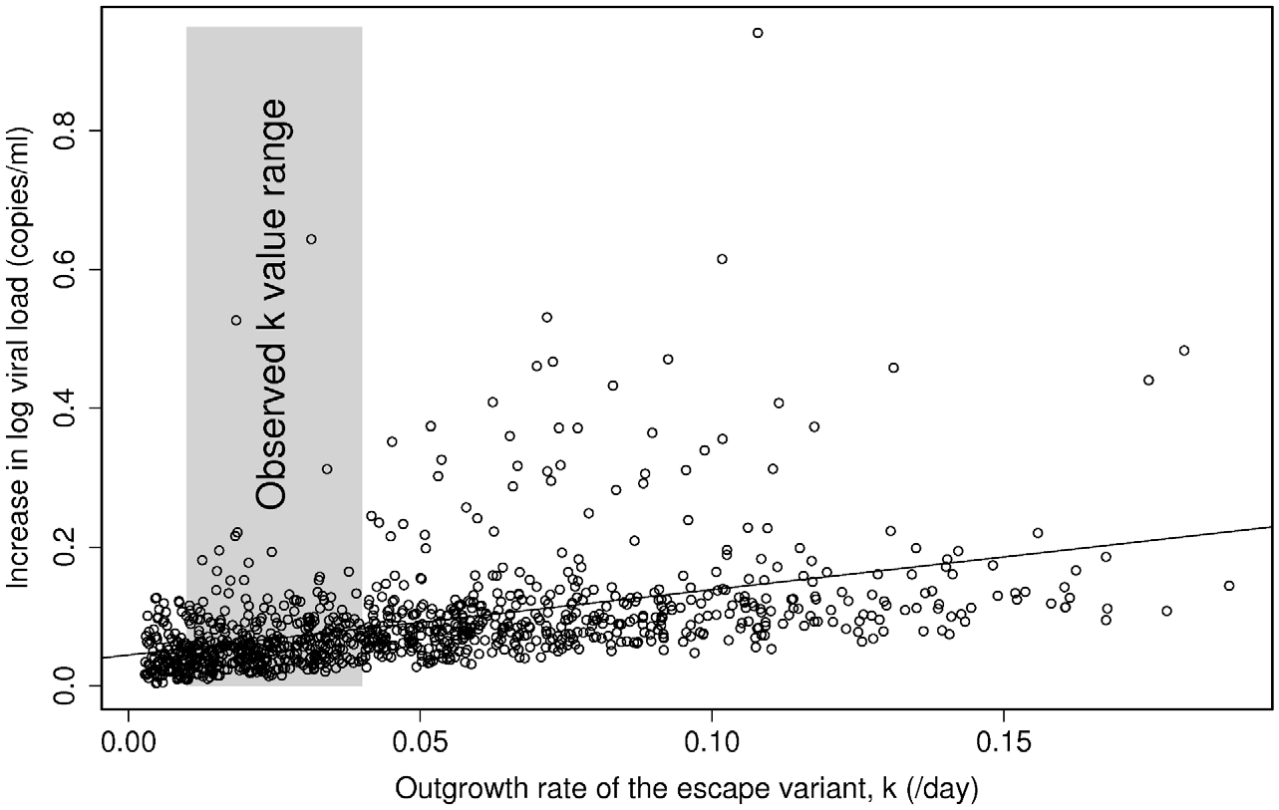
Predicted increase in viral load per escape event as a function of the outgrowth rate of the escape variant. Results from the first 1,000 runs from the mathematical model are plotted. The increase in viral load after an escape event is significantly positively correlated with the outgrowth rate (k) of the escape variant (Spearman's rank correlation: p<0.0001, rho = 0.59). The median predicted increase in viral load for observed values of the outgrowth rate (k, shaded area) is approximately 0.051 log (95% confidence interval: 0.050–0.052).

The variant was assumed to have reduced replicative ability compared to the wildtype (i.e. although the variant is fitter in the presence of a CTL response, it is less fit in the absence). The system was modelled under the “worst case” scenario that the CTL response is unable to target the escaped epitope again and so does not regain control of viraemia. In all 100,000 runs of the model, covering a wide range of biologically plausible parameters, there was an increase in viral load upon escape. The increase in viral load upon escape was significantly correlated with the outgrowth rate of the variant (Spearman's rank correlation: p<0.0001; rho = 0.59). For outgrowth rates representative of what is observed in chronic HIV infection (i.e. the variant replaces the wildtype at rate 0.01–0.04 per day, corresponding to variant outgrowth in 230–900 days [4], [8], [13], [15], [16], [20], [27]–[31]), the median increase in viral load was 0.051 log copies ml^−1^ (95% confidence interval: 0.050–0.052; Figure 1; Figure S1).

A second scenario, in which the infection and virion production rates of the variant were set independently of the wildtype such that the variant was permitted to be fitter than the wildtype at the time of escape, was also considered. In this model, the median increase in viral load was 0.050 log copies ml^−1^ (95% confidence interval: 0.044–0.056). Even if the outgrowth rate of the escape variant was an order of magnitude higher (e.g. 0.4 per day, corresponding to the variant replacing the wildtype in 23 days), the median predicted increase in viral load was still only 0.27 log. As before, there was a significant positive correlation between the outgrowth rate of the escape variant and the resulting increase in viral load (Spearman's rank correlation: p<0.0001; rho = 0.59). Interestingly, for this model there was a fraction (12%) of cases in which escape resulted in a decrease in viral load.

To test the robustness of the prediction that escape leads to a small increase in viral load, additional mathematical models were simulated which tested different assumptions and explicitly modelled the CTL response as a separate dynamic population (see Table S1 for further details). Median changes in viral load for these models ranged from an increase of 0.017 to 0.037 log copies ml^−1^ (see Text S1 for full details).

### Relationship between number of “escape events” and viral load in a chronically infected cohort

Following Moore *et al.* [11] and Kiepiela *et al.* [32], we define an “escape event” as a coding change in an epitope that is statistically significantly associated with possession of the restricting HLA allele. We introduce two differences into our analysis. Firstly, we cap the number of escape events per epitope at 1 (so that we do not double-count additional mutations required for escape from the same CTL clone). Secondly and more importantly, we adjust for the background level of mutation. Variation in the length of infection and viral replicative rates between individuals are confounding effects which can increase both the number of mutations and viral load making it impossible to infer the impact of HIV escape on viral load simply by studying raw cross-sectional data [22].

In order to correct for the background level of mutation, we performed multivariate regression. This allowed us to test whether the number of escape events (NEE) is a significant independent predictor of viral load after a possible relationship between viral load and the background level of mutation (quantified by NSE, the number of synonymous changes in epitopes per individual) had been taken into account.

**Figure 2:**
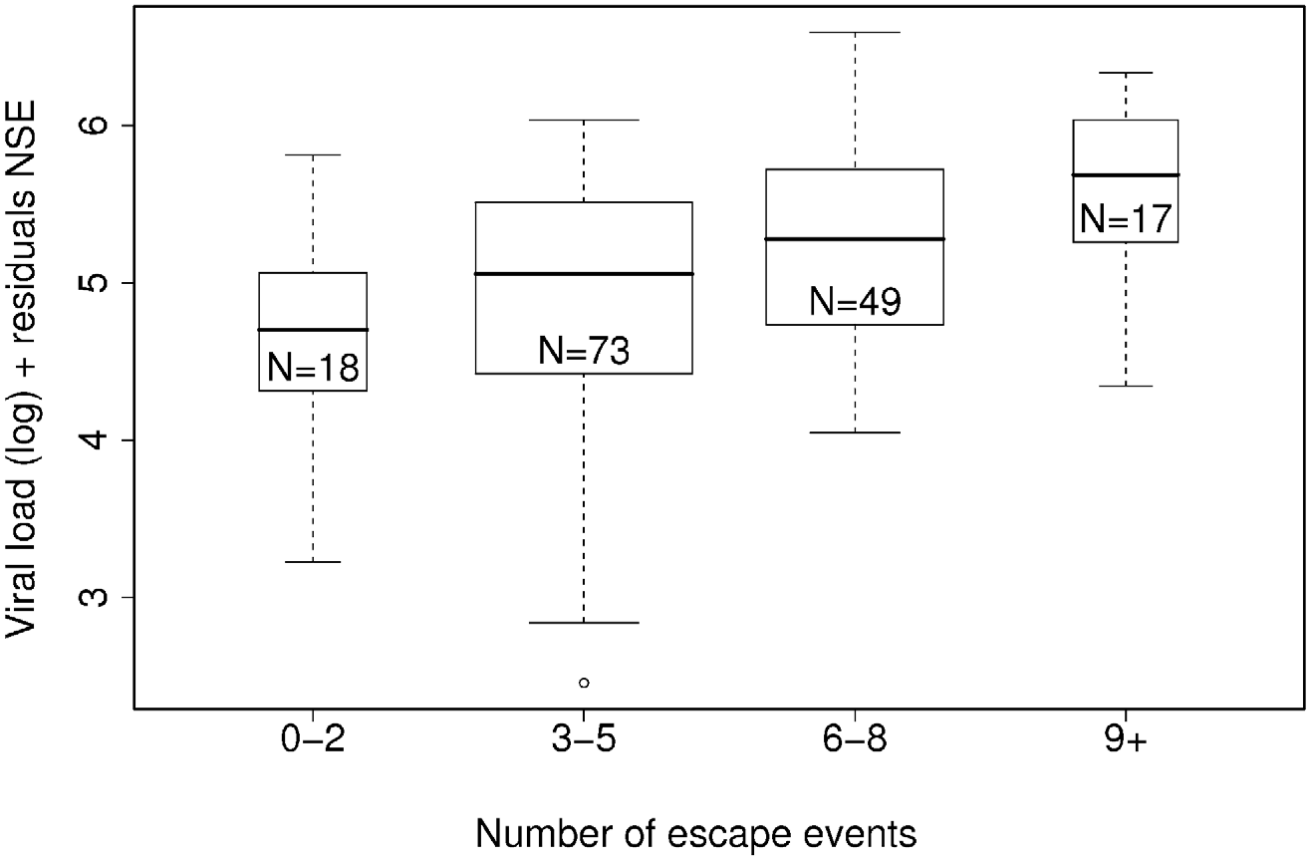
The number of escaped events (NEE) against log viral load, corrected for the number of synonymous epitopes (NSE). Number of escape events is a significant predictor of log viral load after the background level of mutation is taken into account (multiple linear regression: p = 0.0021, gradient = 0.11, r^2^ = 0.060, N = 157). The width of each bar is proportional to the number of points. Alternate representations of this data are shown in Figure S2.

The analysis showed that the number of escape events is a significant independent predictor of log viral load when the number of synonymous changes is taken into account (Figure 2, multiple linear regression: two-tailed p = 0.0021, gradient = 0.11, r^2^ = 0.060; Figure S2 shows alternative representations; linear regression of NEE alone on log viral load: p = 0.029, gradient = 0.055). Neither changing the definition of “escape event” to include only mutations with a decrease in predicted binding affinity (multiple linear regression: p-value = 0.42, gradient = 0.13; see Text S1), nor stratifying the cohort based on frequently occurring alleles (see Table S2), altered these results. Repeating this analysis with CD4+ T-cell count and the number of escape events showed a trend toward a negative association (multiple linear regression: two-tailed p = 0.062, gradient = −23).

In order to investigate the relationship between escape from protective alleles and viral load, we performed three analyses. First, we removed individuals with protective alleles from the multiple linear regression (see Table S2). The “unprotected” group still has a statistically significant positive association between log viral load and NEE (multiple linear regression p = 0.00024, coef = 0.19), although no association was observed in the “protected” group (p = 0.20, coef = 0.060). Second, we added the number of protective alleles and the number of not-protective alleles possessed by an individual as (two) additional predictors of log viral load to the multiple linear regression. We find that all four factors are significant independent predictors of log viral load (multiple linear regression: NEE p = 0.000444, coef = 0.12038; NSE p = 0.009526, coef = −0.09110; Protective p = 0.006509, coef = −0.47809; Not-protective p = 0.006715, coef = 0.28016). Third, we wanted to further explore whether alleles associated with a lower viral load were protective because they presented epitopes where escape was rare. To do this we calculated the NEE of epitopes presented by protective alleles by individual and compared it to the NEE of epitopes presented by not-protective alleles. To ensure equal sample size in the “protective” and “not-protective” groups, we selected a number of alleles in either group such that 10% of the cohort possessed a protective allele, and 10% of the cohort possessed a not-protective allele. We observe that epitopes restricted by protective HLA alleles had a median of 1.0 additional escape events than not-protective alleles (Wilcoxon rank sum test: p = 0.013, difference = 1.0). These three pieces of analysis show that escape in epitopes presented by not-protective HLA class I alleles is detrimental for the host, and that protective HLA class I alleles do not have fewer escape events than ‘detrimental’ alleles. The “protectiveness” of an allele is therefore not attributable to an inability to escape such responses.

To check for robustness, we performed a bootstrap analysis by sampling 10,000 times with replacement from the Full cohort. We found that 92% of the bootstrap runs resulted in a significant positive association between log viral load and the number of escape events, the median two-tailed p-value of all runs was 0.0019 (95% confidence interval: 0.0018–0.0021; Figure S3A).

### Quantifying the impact of an “escape event” on viral load

By calculating the gradient of the line of best fit through the data, after correcting for the background level of mutation, we find that each escape event causes a median increase in viral load of 0.11 log copies ml^−1^ (95% confidence interval: 0.040–0.18), or an absolute median increase of 8,500 copies ml^−1^. Overall, the number of escape events could only explain about 6% of the variation in viral load in the cohort.

### Mutations in *pol* account for the majority of the impact of escape on viral load

**Figure 3:**
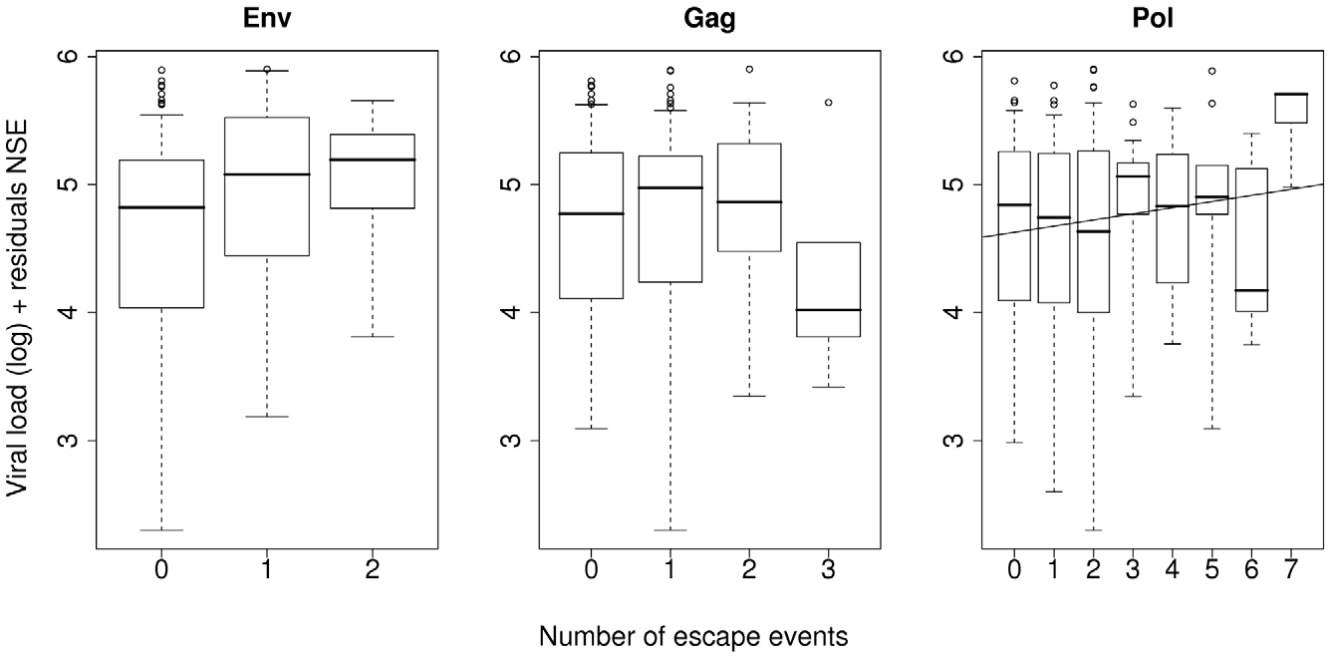
The number of escaped events (NEE) against log viral load, corrected by the number of synonymous epitopes (NSE), stratified by gene. Data used in Figure 2 was stratified by each HIV gene and the multiple linear regression repeated. Only escape events in *pol* were statistically significant independent predictors of log viral load (p = 0.0059, gradient = 0.14, line of best fit drawn), escape events in all other genes were not significant (data not plotted for genes *nef*, *rev*, *vif* and *vpr*, see Table S3).

Next we investigated whether escape events in any particular gene were associated with the increase in viral load, so we repeated the multi-linear analysis on each gene independently (Figure 3; Table S3). We found a significant positive association between log viral load and coding changes in *pol* (multiple linear regression: two-tailed p-value = 0.0059, gradient = 0.14), but not in any other gene. Additionally, a significant positive association was found between viral load and escape events in *pol* in 83% of bootstrap runs (Figure S4 and Table S4). Amongst the other genes, we noted that there was a **decrease** in log viral load with escape events in *gag*, although this was not statistically significant (multiple linear regression: two-tailed p-value = 0.91, gradient = −0.011; Table S3). As Gag-specific CTL responses have been shown as protective [33]–[37], we had expected escape to lead to a large rise in viral load. To check that power was not an issue, we repeated the analysis using a longer HLA-gag epitope list derived using a phylogenetically corrected method [38]. This confirmed the negative association between escape events in *gag* and log viral load (multiple linear regression: two-tailed p-value = 0.013, gradient = −0.091).

Next, we investigated which gene (or genes) was driving the effect in the 92% of bootstrap runs that gave a significant association between the total number of escape events in all genes and viral load (see previous section “Relationship between number of escape events and viral load”). This analysis showed that escape events in most genes were detrimental for the host, but that escape events in Pol epitopes were most frequently (81%) behind the increase in viral load (Table S5).

**Figure 4:**
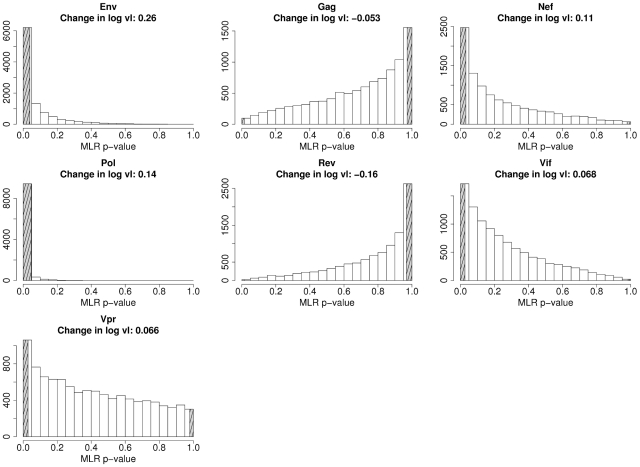
Bootstrap distribution of one-tailed p-values from multiple linear regression of each gene in the K37 epitope list. Subjects were sampled with replacement 10,000 times from the Extended cohort (N = 347). The shaded area on the left-hand side of each graph indicates the proportion of runs with a statistically significant increase in log viral load (one-tailed p≤0.025), and the shaded area on the right indicates a significant decrease in log viral load (one-tailed p≥0.975). In each panel the median change in log viral load associated with an escape event in that gene is presented.

Finally, we investigated individual gene effects in the Extended cohort (N = 347) in order to increase power. We could not study this cohort in the previous analysis as we did not have sequence data for all genes for all individuals; however this is unnecessary for the purposes of individual gene analysis. As the Full cohort is a subset of the Extended cohort, the results must not be considered independent. Analysis of this extended cohort strengthened and extended our previous results. Escape events in *pol* were still significantly associated with an increase in viral load (at a more significant two-tailed p-value of 0.0032). The results of a bootstrap run on this extended cohort are shown in Figure 4. Genes appear to fall in one of two distinct groups: genes in which escape events were associated with a high viral load (*env*, *nef*, *pol* and *vif*) and genes in which escape events were associated with a low viral load (*gag* and *rev*).

## Discussion

To date, it has not been possible to quantify the impact of HIV-1 escape on viral load. Longitudinal datasets are too sparse to allow firm conclusions and studies of cross-sectional datasets have been impossible due to confounding factors. Furthermore, previous studies [39]–[41] have concentrated on escape from CTLs associated with protection, e.g. Gag-specific CTLs, or on protective alleles, e.g. B*57, which although interesting and important for determining mechanisms of protection, are unlikely to be representative and may give a distorted view of the importance of escape. Here, we have investigated the impact of “typical” (i.e. average) escape events in seven genes in chronic HIV-1 infection using two independent methods: mathematical modelling and cross-sectional cohort analysis, using multiple regression to remove confounding factors.

Firstly, we simulated the emergence of HIV-1 escape variants using an extension of the Perelson/De Boer model of HIV-1 dynamics [26], using parameters selected from a wide range of biologically realistic values. We modelled the system under the “worst case scenario” that the CTL response does not regain control of viraemia following escape. This modelling showed that the increase in viral load per escape event was significantly positively correlated with the outgrowth rate of the variant (Spearman's rank correlation: p<0.0001; rho = 0.59). The median increase in viral load was 0.051 log copies ml^−1^ per escape event. Adapting the model to allow CTLs to partially regain control would lead to a smaller long-term increase in load per escape event. A range of alternative models based on different assumptions led to similar conclusions.

Secondly, we analysed a cross-sectional cohort of 157 HIV-1 C-clade infected individuals. We used a novel method to remove confounding factors which permits the analysis of data-rich cross-sectional cohorts. By treating the number of synonymous changes accrued by an individual as a clock that keeps count of the number of “mutation events”, we corrected for differences in individual mutation rates, regardless of whether the increased mutation frequency resulted from a higher viral set point, higher viral replication rate or longer length of infection (and hence lower CD4+ count). We show a statistically significant positive association between the number of mutated epitopes and viral load. We found a small increase in viral load of 0.11 log per escape event. To put this into context, the median standard deviation of random fluctuations in log viral load over time in a chronically HIV B-clade infected cohort is 0.32 (see Text S1). The association between the number of mutated epitopes and viral load was mainly accounted for by mutations in *pol*. The escape events studied could have occurred at any time point prior to sampling, i.e. evolved during the acute [42] or chronic phases, or were already present in the transmitted infecting virus.

The small increase in viral load per escape event that we observed in the cohort could be explained by two (non-exclusive) hypotheses. Firstly, the impact of escape on viral load is transient due to the flexibility of the CTL response which adapts to recognise either the escape variant or a previously sub-dominant epitope, resulting in renewed and long-term suppression of viral load. Secondly, the impact of escape is small because the variant has a slow outgrowth rate (i.e. a small net fitness advantage compared with the wildtype), so the accompanying increase in viral load is not large. Under the first hypothesis, escape initially causes a large increase in viral load but then the CTL response adapts and partially regains control of viraemia, either by targeting previously unrecognised or sub-dominant epitopes [16], [43], [44] or by developing a new CTL response against the variant in the case of escape due to loss of TCR recognition [45]. The transient nature of the increase would unlikely be reflected in a cross-sectional study, and so the observed increase in viral load would be small. Under the second hypothesis, the increase in viral load per escape event is small simply because the outgrowth rate of the escape variant is slow (i.e. CTL selection pressure on the wildtype minus fitness cost of the variant is small). The mathematical modelling we performed allowed us to test these two hypotheses. For outgrowth rates observed in chronic HIV-1 infection [4], [13], [15], [16], [20], [27]–[31], equivalent to a variant replacing the wildtype in 230 to 900 days from first appearance of the variant, we found that the median increase in viral load was predicted to be around 0.051 log copies ml^−1^. Whilst we do not rule out that existing CTL responses are strengthened or that new CTL responses can arise to regain control of viraemia after an escape event, this mechanism is unnecessary to explain the observed modest increase in viral load per escape event. Rather, the small increase in viral load is exactly what would be predicted given the observed outgrowth rate of escape variants in late primary and chronic infection.

Additionally, the modelling showed that the increase in viral load per escape event was significantly positively correlated with the outgrowth rate of the variant: that is, the faster the variant outgrows the wildtype, the larger the resulting increase in viral load. This is consistent with the observation that protective HLA class I alleles associated with slow progression to AIDS tend to present epitopes where escape variants have a slower outgrowth rate [46]. These two observations predict, somewhat counter-intuitively, that escape from protective HLA alleles will result in a smaller increase in viral load than escape from non-protective alleles.

A similar argument could explain our finding that escape events in *gag* did not cause a measurable increase in viral load. Given the protective role of Gag-specific CTLs [33]–[37], it might be expected that escape from these CTLs would be particularly detrimental. However, some escape variants in *gag* have also been shown to carry very heavy fitness costs [47]–[49]. Consequently, the outgrowth rate of escape variants in *gag* will not necessarily be fast. In fact, recent preliminary data suggests the opposite: that escape variants in *gag* have a significantly slower outgrowth rate than variants in other proteins [46]. This would explain why escape mutations in *gag* have not been found to lead to large increases in viral load. Results from the modelling suggest one potential mechanism how escape can lead to a lower viral load. Intuitively, we would expect that variants must have a net fitness advantage over the wildtype, which results in a higher viral load upon escape. However, in Model 2, we observed that 12% of all runs resulted in a decrease in viral load and the outgrowth of the variant. Our analysis of these runs showed that in all cases, the variant was more infectious than the wildtype (higher β′) but able to produce fewer virions (lower h′), resulting in an increase in the number of variant-infected cells but not an increase in the viral load. This intriguing result suggests that although the variant has a net fitness advantage over the wildtype, it may only be manifest at the level of infected cells and not necessarily at the level of free virus. It is therefore not unfeasible for an ‘attenuated variant’ to emerge. A prediction of the model is that proviral load would always be higher after escape. Alternatively, the negative association between escape events in *gag* and viral load may simply be due to individuals with protective HLA class I alleles, which are associated with lower viral load, mounting more Gag-specific CTL responses and therefore the emergence of more escape variants in *gag*.

A number of studies have reported a relationship between protective HLA class I alleles and low levels of viral escape, suggesting that escape is an important determinant of progression to AIDS [46], [50]–[52]. However, we did not find any evidence that protective HLA class I alleles were associated with lower levels of viral escape. Instead, we observed that individuals possessing a protective allele had more escape events than individuals with an allele associated with a higher viral load. The protectiveness of an allele therefore appears unrelated to its ability to prevent viral escape (despite the high fitness cost of escape mutations), but rather suggests that protective alleles present epitopes where variants only have a small net fitness advantage.

We find that escape only determines approximately 6% of variation in viral load. How can we reconcile the low impact of escape events on viral load with disease progression, given the strong association between HLA and disease progression? As viral load is subject to high levels of random fluctuations within an individual over time, one possible explanation is that viral load is not always strongly associated with progression [53]. However, we also failed to find a strong association between escape events and CD4^+^ T cell count, which is usually a better correlate of progression. Another possible explanation is that HLA-mediated protection may not be completely dependent on CTL, as other HLA-mediated factors could play a greater role in HIV infection, e.g. NK cells or linkage disequilibrium with a protective gene. It is therefore entirely plausible that CTL escape only partially contributes to HLA-mediated protection. Furthermore, a much greater number of CTL-independent factors potentially determine viral load, for example, CCR2 polymorphisms and CCR5 deletions [54]–[56], Nef deletions [57], NK cell receptor polymorphism [58], viral tropism [59] and host activation status [60]. A recent study also showed significant genetic and immunological heterogeneity between people who naturally control HIV-1 viremia [33]. Other studies have estimated that HLA class I type determines about 3%–15% of viral load [33], [61]–[63] and that escape mutations determine about 30% of HLA class I protection [46]. If we assume in the most extreme case that that HLA-associated protection is solely due to the ability to target epitopes where escape is rare, then this would indicate that only around {3%–15%}×30% = 0.9% to 4.5% of variation in viral load could be explained by escape, which is very similar to our estimate of 6%. Given the large number of possible determinants of viral load it is perhaps not surprising that HIV escape from CTL can only explain a small proportion of an individual's viral load.

We predicted mathematically and also showed by analysis of an HIV-1 infected cohort that HIV escape from the CTL response is associated with a small increase in viral load. Although the finding that a typical escape event has a modest impact on viral load is surprising, it is consistent with firstly, the failure to find a clear association between escape and viral load in longitudinal studies [18]–[21], secondly, the low outgrowth rate of escape variants in chronic infection [8], and lastly, the weak positive correlation between uncorrected viral load and HLA-associated polymorphisms in *gag* observed by Brumme *et al.* [23].

This study shows that although the impact of HIV-1 escape from CTLs is highly statistically significant, the effect in clinical terms is mild: an increase in viral load of 0.11 log copies ml^−1^ will have few consequences for patient health [64]. This unexpected result demands further independent studies to corroborate it. If replicated, this result would suggest that, with the crucial exception of vaccine design, the focus on HIV escape may be out of proportion to its importance with other factors playing a more significant role in determining viral load in chronic HIV-1 infection.

## Materials and Methods

### Mathematical model of viral escape

The Perelson/De Boer model of HIV-1 dynamics [26] was adapted to include HIV-1 escape variants, which have escaped a single CTL response (Equation 1).
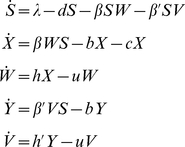
(1)The five populations (all measured per mm^3^) are as follows: uninfected CD4^+^ cells (S); CD4^+^ cells infected with wildtype virus (X); free wildtype virus (W); CD4+ cells infected with variant virus (Y); free variant virus (V). Parameters are described in Table 1. The model was simulated using *lsoda* in R. Each run sampled parameters from ranges obtained from the literature [65]–[71], and the variant was assumed to be attenuated compared to the wildtype, such that β>β′ and h>h′. The median outgrowth rate of the variant (k, see below) observed in chronic HIV-1 infection [4], [13], [15], [16], [20], [27]–[31] is 0.01 d^−1^ to 0.04 d^−1^ [8]; we were interested in typical escape events and so runs with an outgrowth rate outside this range were not included in the analysis and the model was run until 100,000 simulations had been accumulated. From these runs the difference in viral load of the wildtype (W+V at t_0_) and the variant (W+V at t_end_) was calculated. A full description of this model, as well as other models considered, can be found in Text S1.

**Table 1:** Parameter ranges, descriptions and references for the mathematical model.

Parameter	Range	Description (units)	Reference
λ	5–30	Influx of new uninfected CD4^+^ cells (c d^−1^)	[65]
d	0.013–0.078	Natural death rate of uninfected CD4^+^ cells (d^−1^)	[66]
β	0.001–0.01	Infection rate of wildtype virus (v^−1^ d^−1^)	[65]
β′	0.001–0.01	Infection rate of variant virus (v^−1^ d^−1^)	[65]
b	0.5–1.0	Death rate of infected CD4^+^ cells from all other sources (d^−1^)	[65], [67]
c	0.02–0.2	Death rate of CD4^+^ cells by a single CTL clone (d^−1^)	[8], [68]
h	20–200	Burst rate of CD4^+^ cells infected with wildtype (v c^−1^ d^−1^)	[69]
h′	20–200	Burst rate of CD4^+^ cells infected with variant (v c^−1^ d^−1^)	[69]
u	3–300	Clearance rate of free virus (d^−1^)	[70], [71]

Note that parameter b is the rate of death of infected CD4^+^ cells attributable to CTL-independent death and all CTL responses other than the single CTL response which is escaped (parameter c). Units c: cells mm^−3^; v: virions mm^−3^; d: day.

### Definition of k: The outgrowth rate

The outgrowth rate (net growth advantage) of an escape variant is the rate at which the variant outgrows the wildtype virus. It is defined as the growth rate of the variant minus the growth rate of the wildtype [8], [72], or: (variant replication rate – variant death rate) – (wildtype replication rate – wildtype death rate). As this quantity is not easily expressed in terms of the model parameters, we use the results of the model simulation and Equation 2 to calculate the outgrowth rate of the variant.
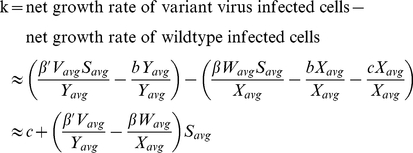
(2)V_avg_ is the average value of variant free virus (V) from the time when the variant first emerges (*t_0_*) to the time when the variant constitutes 99% of the virus population (*t_fix_*). Similar to Asquith *et al.* [8], we do not consider the time for the variant to emerge when calculating the outgrowth rate. *X_avg_* (average number of wildtype-infected cells), *W_avg_* (wildtype free virus), *Y_avg_* (variant-infected cells) and *S_avg_* (uninfected cells) are similarly defined as in Equation 3.
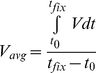
(3)Alternatively, k can be estimated by fitting the solution of a simplified model of escape virus dynamics [8] to the model data using nonlinear least-squares regression (using the Levenberg-Marquardt algorithm, *nls.lm*, in the *minpack.lm* package in R). The two approaches to calculating k are in strong positive agreement (Pearson's product moment correlation coefficient: p<0.0001, r = 0.998). The time for the variant to reach fixation is given by approximately 9/k (see Text S1 for details).

### K37 HLA-epitope list

This list, reproduced in Table S6, is taken from Supplementary Table S3 of Kiepiela *et al.* 2007 [73], and consists of 37 HLA-epitopes pairs which are statistically significantly associated with coding changes in the epitope region (plus three amino acids either side of the epitope region) in individuals with the same HLA class I allele.

### Durban cohort: Full and Extended

Viral RNA sequence, HLA class I genotype and viral load were taken from a treatment naïve HIV-1 C-clade infected cohort attending a prenatal clinic in Durban, as described in [32]. Individuals with a viral load greater than 10^6^ copies ml^−1^ were classed as acutely infected [74], [75] and removed from the analysis. This cohort consisted of two overlapping groups: the Full cohort which consisted of individuals with virus sequence data for all epitopes in the K37 epitope list (N = 157); and the Extended cohort which included all individuals with complete viral sequence data for a single gene (N = 347). The Extended cohort includes all individuals in the Full cohort. All cohort data was collected and anonymised by researchers external to the authors of this paper.

### Definition of HLA-associated polymorphism

Following Moore *et al.* [11] and Kiepiela *et al.* [32], a viral coding change is classed as a ‘HLA-associated polymorphism’ if the variant amino acid is significantly associated with possession of the restricting HLA allele. Polymorphisms will include mutations which disrupt immune surveillance (by altering processing, MHC binding affinity or T-cell recognition), and compensatory mutations which restore fitness to the virus.

### Cohort consensus sequence

For each gene in the HIV-1 genome, all viral amino acid sequences in the cohort were aligned using TCoffee [76], and the most frequent amino acid assigned as the cohort consensus. To obtain the nucleotide consensus, the amino acid sequence was used as an alignment guide for the program *tranalign* (available from http://emboss.sourceforge.net) and the most frequently occurring triplet of nucleotides coding for the most frequent amino acid was chosen as the cohort nucleotide consensus.

### Number of Escape Events (NEE) calculation

The number of epitopes with a coding change (NEE) was calculated for each patient, either across all genes, or by separating the epitopes by gene and calculating a NEE score for each gene. HLA-matched epitopes, plus flanking regions of 3 amino acids either side of the epitope, were taken from the K37 HLA-epitope list and compared to the epitope in the cohort consensus amino acid sequence. Any deviation from the cohort consensus was marked as a putative escape mutation and the number of putative escape events (NEE) calculated. The escape event could have happened at any time point prior to sampling (i.e. during the acute or chronic phases, or was already present in the transmitted infecting virus). The number of escape events per epitope is capped at one, i.e. if there are two HLA-associated polymorphisms in the same epitope then this is counted as a single, and not multiple, escape event.

### Number of Synonymous Epitopes (NSE) calculation

We use NSE to quantify the background level of mutations in an individual. NSE is the equivalent of NEE for synonymous nucleotide changes. The method for obtaining a score for NSE, i.e. epitopes with a silent coding change, is similar to the method for obtaining NEE, save that the individual's viral nucleotide sequence is compared with the cohort consensus nucleotide sequence and only non-coding changes enumerated.

### Correcting log viral load for NSE

To calculate the impact of HLA polymorphisms on viral load after correcting for the “background” number of mutations, we performed multiple linear regression with log viral load as the dependent variable and NEE and NSE as predictors:
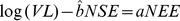
(4)The data plotted in Figures 2 and 3 are partial residual plots, that is, they show viral load after removing the background level of mutation for each individual. The left-hand side of Equation 4 was plotted on the y-axis against NEE on the x-axis, where 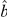 is the regression coefficient for NSE from the fitted full model. The fraction of variation in viral load in the cohort that could be explained by this model was calculated using the R^2^ coefficient.

### Frequency of significant positive association between log viral load and escape events by gene

Patients were selected from the Full cohort at random with replacement 10,000 times. For each bootstrap iteration in which total escape events over all genes was a significant predictor of log viral load, escape events were then split by gene to determine escape events in which gene were significant predictors of log viral load. When multiple genes were found to be significant predictors, we checked if they were independent predictors or correlated with escape events in other genes. Sets of independently significant genes contributing less than 1% of the total runs are grouped together as “Minor effects” and excluded from further analysis. Multiple linear regression was used to determine significance, and the number of synonymous changes was always included as a co-predictor. See Text S1 for full details of the method.

### Fraction of viral load variation explained by HLA class I genotype

To estimate the fraction of viral load variable that can be explained by the HLA class I genotype, we calculated the “Explained Fraction” (EF) metric [61] on data reported by Nelson *et al.* [61], Pereyra *et al.* [33] and Emu *et al.* [62] separately. We categorised the various HLA class I genotypes into two classes: protective alleles and not protective (or susceptible) alleles. Disease states were categorised either using AIDS endpoints (for the Nelson data) or viral load (for Pereyra and Emu data). Methods and results are detailed in full in Text S1.

## Supporting Information

Figure S1 Comparison of viral loads from the mathematical model before and after escape. Each line is the log viral load for a single run from the Attenuated Model, from time zero (before escape) to the end of the model run (after escape). A small amount of random noise was added to the x-axis to increase clarity. Note: 400 of the 10,000 runs were randomly chosen to represent this graph, as plotting all 10,000 runs resulted in an incomprehensible figure. The log viral load is significantly lower after escape (paired t-test: p<0.0001), but the size of the decrease is small (mean of log difference is 0.09, 95% confidence interval: 0.086–0.10).
(0.06 MB TIF)

Figure S2 Comparison of corrected and uncorrected viral load with different measures of sequence variation (c.f. Figure 2 in the main text). Panel A shows the number of escaped epitopes (NEE) against log viral load, corrected for the number of synonymous epitopes (NSE), in cohort of 157 HIV-infected individuals as a graph of points rather than bar graphs. There is a significant positive correlation between the number of escape events and NSE-corrected log viral load (multiple linear regression: p = 0.0000139, r^2^ = 0.12). Panel B shows the number of escaped epitopes (NEE) against log viral load, not corrected for the number of synonymous epitopes (NSE), in cohort of 157 HIV-infected individuals. There is a significant positive correlation between the number of escape events and (uncorrected) log viral load (multiple linear regression: p = 0.026, r^2^ = 0.031). Panel C shows the number of synonymous epitopes (NSE) against log viral load, in cohort of 157 HIV-infected individuals. No statistically significant correlation was found between the number of synonymous epitopes and viral load (multiple linear regression: p = 0.98, r^2^ = 0.00000274).
(0.24 MB TIF)

Figure S3 Bootstrap analysis of the Full cohort, sampled with replacement 10,000 times. Panel A shows the distribution of the one-tailed p-value of NEE as an independent predictor of log viral load (multiple linear regression: median two-tailed p-value was 0.0019, 95% confidence interval 0.0018–0.0021; the shaded area shows the proportion of runs where NEE was significant at a one-tailed p-value of ≤0.025). Panel B shows the distribution of the p-value of NSE as an independent predictor of log viral load (multiple linear regression: median two-tailed p-value was 0.030, 95% confidence interval 0.028–0.031; shaded area shows the proportion of runs where NSE was significant at a one-tailed p-value of ≤0.025). Panel C shows the distribution of the difference in viral load of each of the runs (median log difference: 0.1097; 95% confidence interval 0.1091–0.1104).
(0.26 MB TIF)

Figure S4 Distribution of one-tailed p-values from multiple linear regression of each gene in the K37 epitope list. Subjects were sampled with replacement 10,000 times from the Full cohort (N = 157). The shaded area on the left-hand side of each graph indicates the proportion of runs with a statistically significant increase in log viral load (one-tailed p≤0.025), and the shaded area on the right indicates a significant decrease in log viral load (one-tailed p≥0.975). In each panel the median change in viral load associated with an escape event in that gene is presented. See Table S3 for the percentage of runs by gene where coding changes are statistically significant.
(0.35 MB TIF)

Table S1 Descriptions of the mathematical models. In addition to these models which assumed that the variant virus would always be attenuated compared to the wildtype, an ‘equal fitness’ version was run. Here, the variant was assumed to have the same fitness as the wildtype in the absence of CTL. For these models the infection (β) and virion production (h) rates were the same for wildtype and variant virus. Legend. 5D: five population; 6D: six population; MA: mass action; MM: Michaelis-Menten.
(0.03 MB DOC)

Table S2 Stratification of the Durban cohort by frequent or protective alleles. A frequently occurring allele was defined as occurring in more than 6% of the cohort. An allele was defined as ‘protective’ if the median viral load of all individuals possessing that allele was lower than the median viral load of the cohort, see Figure 3 of Kiepiela *et al.* 2004. For each group, multiple linear regression was performed to determine whether NEE or NSE were significant independent predictors of log viral load.
(0.03 MB DOC)

Table S3 Summary of multiple linear regression on the Full cohort on log viral load against the number of escaped epitopes (NEE), stratified by gene. Results which are statistically significant (two-tailed p<0.05) are shown in bold font.
(0.03 MB DOC)

Table S4 Breakdown by gene the percentage of times escape events predicted log viral load. Percentage of the 10,000 bootstrap runs on the Full cohort where escape events (NEE) in a single gene were a statistically significant predictor of log viral load, independent of the number of synonymous changes (NSE). Also, see Figure S4.
(0.03 MB DOC)

Table S5 Percentage of the 10,000 bootstrap runs on the Full cohort where escape events in a gene were a significant independent predictor of log viral load, stratified by gene and corrected for the number of synonymous changes (NSE). Where there was a statistically significant association between NEE in all genes and log viral load, the genes driving this association were identified using multiple linear regression, or if none had p-values under 0.05, the gene with the lowest p-value in the regression was chosen. N.B. the totals do not sum to 100% as escape events in multiple genes can be significant independent predictors of log viral load.
(0.03 MB DOC)

Table S6 K37 HLA-epitope list. This list is taken from Kiepiela *et al.* (2007), Supplementary Table S3, and consists of 37 HLA-epitopes pairs which are statistically significantly associated with coding changes in the epitope region (plus three amino acids either side of the epitope region) in individuals with the same HLA class I allele.
(0.07 MB DOC)

Text S1 Supplemental information for alternative mathematical models and additional methods and results.
(0.24 MB DOC)
