# Supplementary figures and images for: Quantifying the Impact of Human Immunodeficiency Virus-1 Escape From Cytotoxic T-Lymphocytes
Source: PLoS Comput Biol. 2010 Nov 4;6(11):e1000981. doi: 10.1371/journal.pcbi.1000981 (PMC2973816; doi:10.1371/journal.pcbi.1000981)

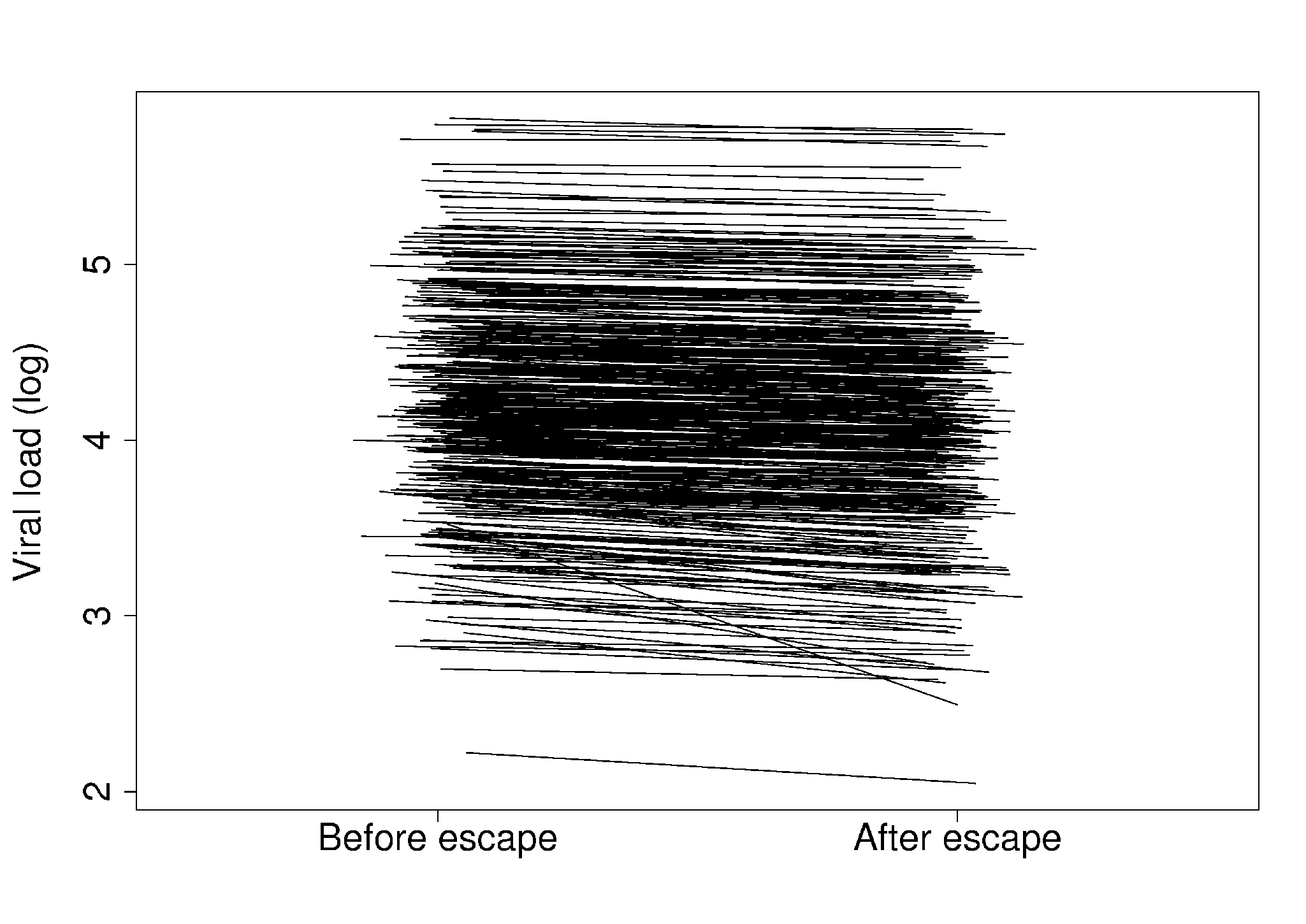

Supplement: Figure S1 — Comparison of viral loads from the mathematical model before and after escape. Each line is the log viral load for a single run from the Attenuated Model, from time zero (before escape) to the end of the model run (after escape). A small amount of random noise was added to the x-axis to increase clarity. Note: 400 of the 10,000 runs were randomly chosen to represent this graph, as plotting all 10,000 runs resulted in an incomprehensible figure. The log viral load is significantly lower after escape (paired t-test: p<0.0001), but the size of the decrease is small (mean of log difference is 0.09, 95% confidence interval: 0.086–0.10). (0.06 MB TIF) [file pcbi.1000981.s001.tif]

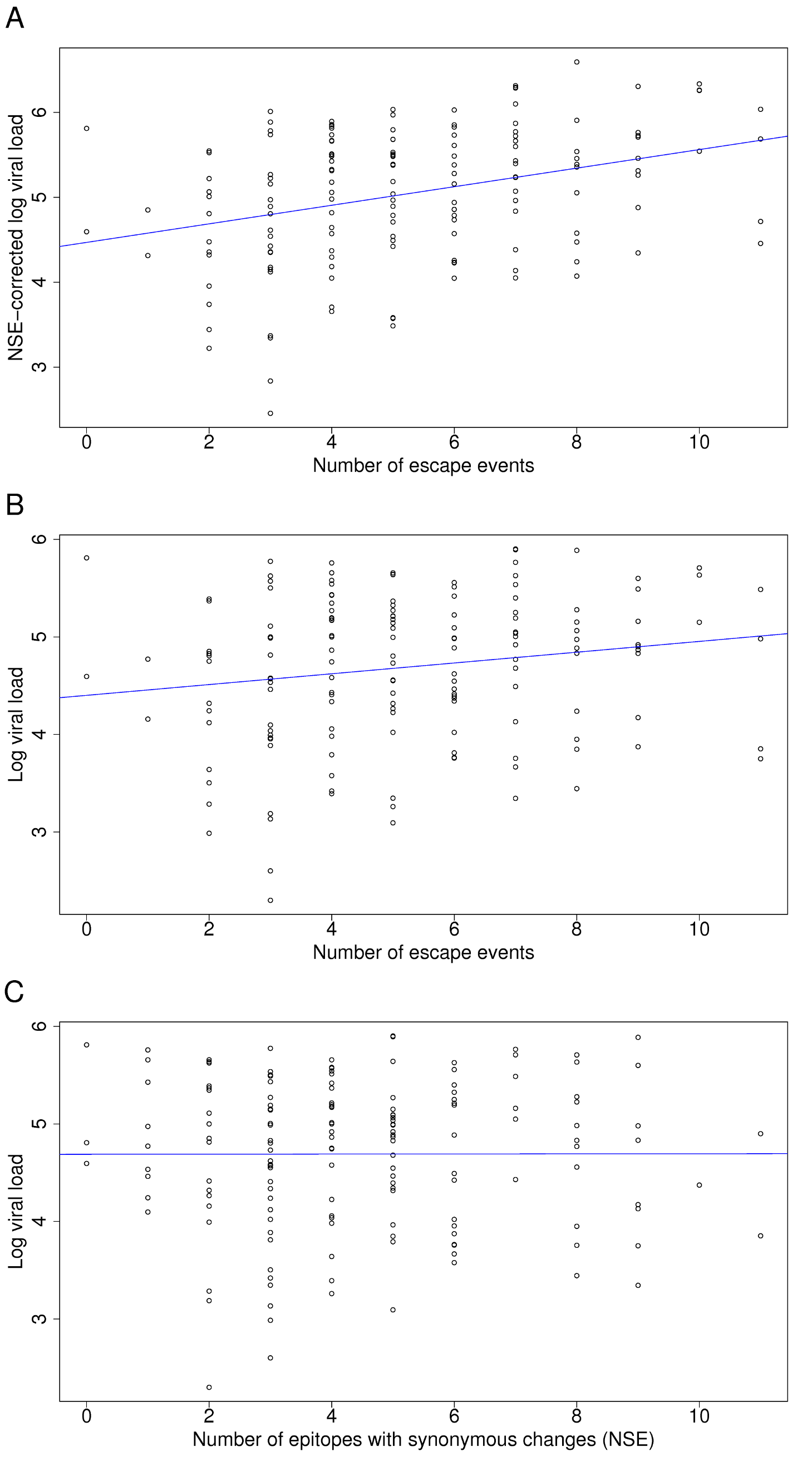

Supplement: Figure S2 — Comparison of corrected and uncorrected viral load with different measures of sequence variation (c.f. Figure 2 in the main text). Panel A shows the number of escaped epitopes (NEE) against log viral load, corrected for the number of synonymous epitopes (NSE), in cohort of 157 HIV-infected individuals as a graph of points rather than bar graphs. There is a significant positive correlation between the number of escape events and NSE-corrected log viral load (multiple linear regression: p = 0.0000139, r2 = 0.12). Panel B shows the number of escaped epitopes (NEE) against log viral load, not corrected for the number of synonymous epitopes (NSE), in cohort of 157 HIV-infected individuals. There is a significant positive correlation between the number of escape events and (uncorrected) log viral load (multiple linear regression: p = 0.026, r2 = 0.031). Panel C shows the number of synonymous epitopes (NSE) against log viral load, in cohort of 157 HIV-infected individuals. No statistically significant correlation was found between the number of synonymous epitopes and viral load (multiple linear regression: p = 0.98, r2 = 0.00000274). (0.24 MB TIF) [file pcbi.1000981.s002.tif]

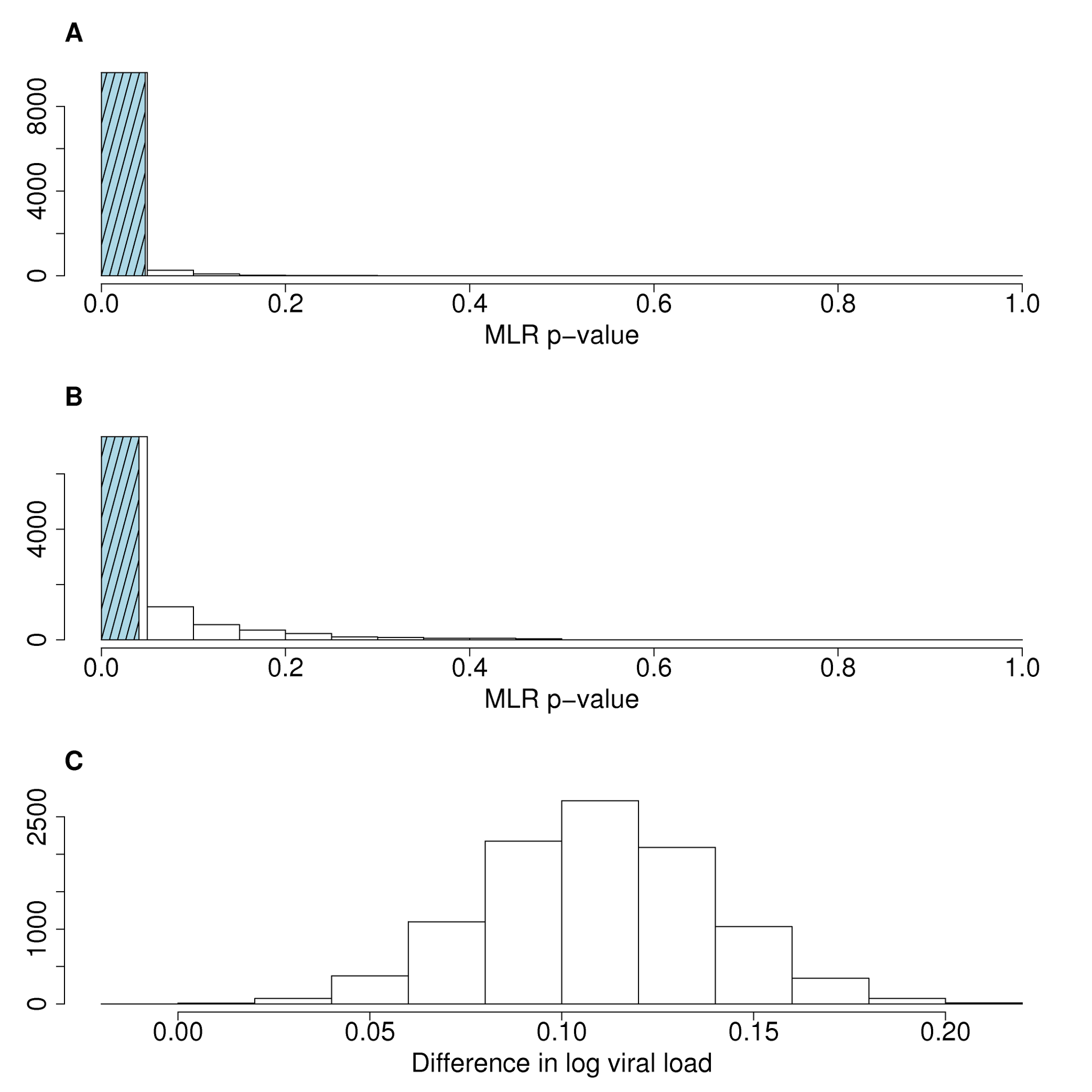

Supplement: Figure S3 — Bootstrap analysis of the Full cohort, sampled with replacement 10,000 times. Panel A shows the distribution of the one-tailed p-value of NEE as an independent predictor of log viral load (multiple linear regression: median two-tailed p-value was 0.0019, 95% confidence interval 0.0018–0.0021; the shaded area shows the proportion of runs where NEE was significant at a one-tailed p-value of ≤0.025). Panel B shows the distribution of the p-value of NSE as an independent predictor of log viral load (multiple linear regression: median two-tailed p-value was 0.030, 95% confidence interval 0.028–0.031; shaded area shows the proportion of runs where NSE was significant at a one-tailed p-value of ≤0.025). Panel C shows the distribution of the difference in viral load of each of the runs (median log difference: 0.1097; 95% confidence interval 0.1091–0.1104). (0.26 MB TIF) [file pcbi.1000981.s003.tif]

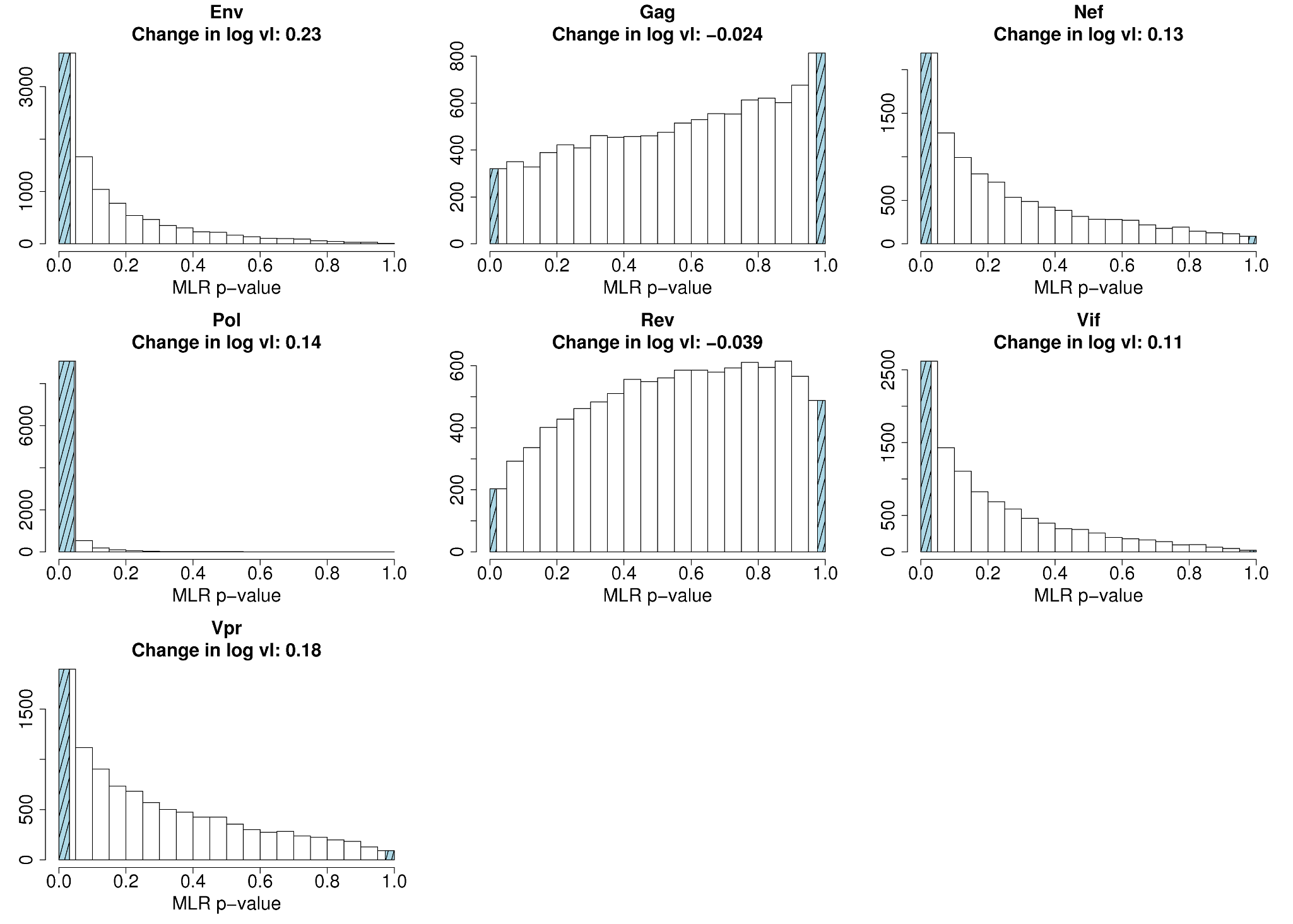

Supplement: Figure S4 — Distribution of one-tailed p-values from multiple linear regression of each gene in the K37 epitope list. Subjects were sampled with replacement 10,000 times from the Full cohort (N = 157). The shaded area on the left-hand side of each graph indicates the proportion of runs with a statistically significant increase in log viral load (one-tailed p≤0.025), and the shaded area on the right indicates a significant decrease in log viral load (one-tailed p≥0.975). In each panel the median change in viral load associated with an escape event in that gene is presented. See Table S3 for the percentage of runs by gene where coding changes are statistically significant. (0.35 MB TIF) [file pcbi.1000981.s004.tif]
