# Supplementary material for: Quantifying the Impact of Human Immunodeficiency Virus-1 Escape From Cytotoxic T-Lymphocytes
Source: PLoS Comput Biol. 2010 Nov 4;6(11):e1000981. doi: 10.1371/journal.pcbi.1000981 (PMC2973816; doi:10.1371/journal.pcbi.1000981)
Supplement: Table S1 — Descriptions of the mathematical models. In addition to these models which assumed that the variant virus would always be attenuated compared to the wildtype, an ‘equal fitness’ version was run. Here, the variant was assumed to have the same fitness as the wildtype in the absence of CTL. For these models the infection (β) and virion production (h) rates were the same for wildtype and variant virus. Legend. 5D: five population; 6D: six population; MA: mass action; MM: Michaelis-Menten. (0.03 MB DOC) [file pcbi.1000981.s005.doc]

| **Model Number** | **Model Description** |
| --- | --- |
| 1 | 5D model with MA terms and attenuated variant |
| 2 | 5D MA model where variant can be fitter than wildtype |
| 3 | 6D model with MA terms |
| 4 | 6D model with MM terms for CTL lysis only |
| 5 | 6D model with MM terms for infection, CTL lysis and CTL proliferation |
| 6 | 6D model with MM terms for CTL lysis and CTL proliferation |
| 7 | 6D model with MM terms for CTL proliferation only |
