# Supplementary material for: Quantifying the Impact of Human Immunodeficiency Virus-1 Escape From Cytotoxic T-Lymphocytes
Source: PLoS Comput Biol. 2010 Nov 4;6(11):e1000981. doi: 10.1371/journal.pcbi.1000981 (PMC2973816; doi:10.1371/journal.pcbi.1000981)
Supplement: Table S2 — Stratification of the Durban cohort by frequent or protective alleles. A frequently occurring allele was defined as occurring in more than 6% of the cohort. An allele was defined as ‘protective’ if the median viral load of all individuals possessing that allele was lower than the median viral load of the cohort, see Figure 3 of Kiepiela et al. 2004. For each group, multiple linear regression was performed to determine whether NEE or NSE were significant independent predictors of log viral load. (0.03 MB DOC) [file pcbi.1000981.s006.doc]

| **Stratify by** | **Number of alleles possessed** | **Number of individuals in group** | **NEE as a significant predictor of viral load** | |
| --- | --- | --- | --- | --- |
|  |  |  | ***P-value*** | **Change in log viral load** |
| Frequently occurring alleles | at least one | 99 | 0.0078 | 0.11 |
|  | none | 58 | 0.14 | 0.11 |
| Protective allele | at least one | 85 | 0.20 | 0.060 |
|  | none | 72 | 0.00024 | 0.19 |
