# Supplementary material for: Quantifying the Impact of Human Immunodeficiency Virus-1 Escape From Cytotoxic T-Lymphocytes
Source: PLoS Comput Biol. 2010 Nov 4;6(11):e1000981. doi: 10.1371/journal.pcbi.1000981 (PMC2973816; doi:10.1371/journal.pcbi.1000981)
Supplement: Table S3 — Summary of multiple linear regression on the Full cohort on log viral load against the number of escaped epitopes (NEE), stratified by gene. Results which are statistically significant (two-tailed p<0.05) are shown in bold font. (0.03 MB DOC) [file pcbi.1000981.s007.doc]

| **Gene** | **Multiple Linear Regression** | |
| --- | --- | --- |
|  | **p-value (two-tailed)** | **Log difference in viral load** |
| **Env** | 0.19 | 0.22 |
| **Gag** | 0.91 | -0.011 |
| **Nef** | 0.37 | 0.12 |
| **Pol** | **0.0059** | **0.14** |
| **Rev** | 0.85 | -0.042 |
| **Vif** | 0.30 | 0.11 |
| **Vpr** | 0.45 | 0.18 |
