# Supplementary material for: Quantifying the Impact of Human Immunodeficiency Virus-1 Escape From Cytotoxic T-Lymphocytes
Source: PLoS Comput Biol. 2010 Nov 4;6(11):e1000981. doi: 10.1371/journal.pcbi.1000981 (PMC2973816; doi:10.1371/journal.pcbi.1000981)
Supplement: Table S4 — Breakdown by gene the percentage of times escape events predicted log viral load. Percentage of the 10,000 bootstrap runs on the Full cohort where escape events (NEE) in a single gene were a statistically significant predictor of log viral load, independent of the number of synonymous changes (NSE). Also, see Figure S4. (0.03 MB DOC) [file pcbi.1000981.s008.doc]

| **Gene** | **Percentage of statistically significant runs (%)** | |
| --- | --- | --- |
|  | **Increase in viral load** | **Decrease in viral load** |
| **Env** | 25 | 0 |
| **Gag** | 2 | 4 |
| **Nef** | 14 | 0 |
| **Pol** | 83 | 0 |
| **Rev** | 1 | 2 |
| **Vif** | 16 | 0 |
| **Vpr** | 12 | 0 |
