# Supplementary material for: Quantifying the Impact of Human Immunodeficiency Virus-1 Escape From Cytotoxic T-Lymphocytes
Source: PLoS Comput Biol. 2010 Nov 4;6(11):e1000981. doi: 10.1371/journal.pcbi.1000981 (PMC2973816; doi:10.1371/journal.pcbi.1000981)
Supplement: Table S5 — Percentage of the 10,000 bootstrap runs on the Full cohort where escape events in a gene were a significant independent predictor of log viral load, stratified by gene and corrected for the number of synonymous changes (NSE). Where there was a statistically significant association between NEE in all genes and log viral load, the genes driving this association were identified using multiple linear regression, or if none had p-values under 0.05, the gene with the lowest p-value in the regression was chosen. N.B. the totals do not sum to 100% as escape events in multiple genes can be significant independent predictors of log viral load. (0.03 MB DOC) [file pcbi.1000981.s009.doc]

| **Gene** | Env | Gag | Nef | Pol | Vpr | Vif | Rev |
| --- | --- | --- | --- | --- | --- | --- | --- |
| **Significant (%)** | 22 | 2 | 12 | 81 | 11 | 15 | 1 |
