# Supplementary material for: Quantifying the Impact of Human Immunodeficiency Virus-1 Escape From Cytotoxic T-Lymphocytes
Source: PLoS Comput Biol. 2010 Nov 4;6(11):e1000981. doi: 10.1371/journal.pcbi.1000981 (PMC2973816; doi:10.1371/journal.pcbi.1000981)
Supplement: Table S6 — K37 HLA-epitope list. This list is taken from Kiepiela et al. (2007), Supplementary Table S3, and consists of 37 HLA-epitopes pairs which are statistically significantly associated with coding changes in the epitope region (plus three amino acids either side of the epitope region) in individuals with the same HLA class I allele. (0.07 MB DOC) [file pcbi.1000981.s010.doc]

| **Gene** | **HLA class I allele** | **Sequence** | **HXB2 start** | **HXB2 end** |
| --- | --- | --- | --- | --- |
| Env | A*29 | SFDPIPIHY | 209 | 217 |
| Env | C*06:02 | AKTIIVHL | 272 | 288 |
| Gag | A*29 | LYNTVATLY | 78 | 86 |
| Gag | A*23:01 | HYMLKHIVW | 28 | 36 |
| Gag | B*57:03 | KAFSPEVIPMF | 162 | 172 |
| Gag | B*57:03 | TSTLQEQIAW | 240 | 249 |
| Gag | B*39:10 | TPQDLNTML | 180 | 188 |
| Gag | B*58:01 | TSTLQEQIAW | 240 | 249 |
| Gag | B*35 | PPIPVGDIY | 254 | 262 |
| Gag | B*07:02 | GPGHKARVL | 355 | 363 |
| Gag | B*81:01 | TPQDLNTML | 180 | 188 |
| Gag | C*03:04 | YVDRFFKTL | 296 | 304 |
| Gag | B*08:01 | NPDCKTIL | 317 | 334 |
| Nef | C*07:01 | KRQEILDLWVY | 105 | 115 |
| Nef | A*23:01 | RYPLTFGW | 134 | 141 |
| Nef | B*18 | QEILDLWVY | 104 | 121 |
| Pol | B*44:03 | QEEHEKYHSNW | 722 | 738 |
| Pol | B*42:01 | KIIKDYGKQM | 973 | 990 |
| Pol | B*42:01 | LPPIVAKEI | 743 | 751 |
| Pol | B*15:10 | THLEGKVIL | 781 | 789 |
| Pol | B*15:03 | RKVKIIKDY | 978 | 986 |
| Pol | B*15:03 | FKRKGGIGGY | 900 | 909 |
| Pol | B*15:03 | IQQEFGIPY | 850 | 858 |
| Pol | A*03:01 | ILKLAGRWPVK | 816 | 826 |
| Pol | A*30:02 | KIQNFRVYY | 934 | 942 |
| Pol | B*81:01 | SPIETVPVKL | 158 | 167 |
| Pol | B*58:01 | IALESIVIW | 530 | 538 |
| Pol | B*42:01 | YPGIKVRQL | 426 | 434 |
| Pol | B*35 | FSVPLDEGF | 264 | 281 |
| Pol | B*18 | NPEIVIYQY | 330 | 338 |
| Pol | B*18 | NETPGIRYQY | 292 | 301 |
| Pol | A*03:01 | RVYLSWVPAHK | 685 | 695 |
| Rev | B*15:10 | IHSISERIL | 52 | 60 |
| Vif | B*42:01 | HPKVSSEVHI | 48 | 57 |
| Vif | B*15:10 | WHLGHGVSI | 79 | 87 |
| Vif | A*68:01 | EVHIPLGEAR | 48 | 65 |
| Vpr | A*68:02 | ETYGDTWTGV | 48 | 57 |
