# Supplementary material for: Quantifying the Impact of Human Immunodeficiency Virus-1 Escape From Cytotoxic T-Lymphocytes
Source: PLoS Comput Biol. 2010 Nov 4;6(11):e1000981. doi: 10.1371/journal.pcbi.1000981 (PMC2973816; doi:10.1371/journal.pcbi.1000981)
Supplement: Text S1 — Supplemental information for alternative mathematical models and additional methods and results. (0.24 MB DOC) [file pcbi.1000981.s011.doc]

**Quantifying the Impact of Human Immunodeficiency Virus-1 Escape From Cytotoxic T-Lymphocytes**

Supplemental Text

1. Supplemental Mathematical Models: Methods and Results: p1

2. Supplemental Methods: p10

3. Supplemental References: p18

Supplemental Mathematical Models: Methods and Results

Descriptions of the additional mathematical models are summarised in Table S1.

2D Model

This is the simplified virus escape dynamics model as described in Asquith *et al.* 2006 [1]. The actual values of k in the 5D model (where k was calculated from the parameters and average values of the populations between time 0 and time at fix, see Equation 2 in the main text) were in very good agreement with the fitted estimate of k in the 2D model (where k was obtained by fitting the proportion of variant over time between time zero and time to fix to the model given in Equation S4). We did not explicitly run the 2D model.

5D Model

This model is described by Equation 1 in the main text. Uninfected CD4^+^ cells (S) are produced at constant rate λ, die naturally at rate d and are infected at rate β by free wildtype virus (W) to produce wildtype-infected cells (X), or rate β' by free variant virus (V) to produce variant-infected cells (Y). Infected cells (wildtype and variant) die at rate b (this rate incorporates natural death, viral toxicity, CTL-mediated lysis by CTLs recognising epitopes other than the one in which escape occurred, and non-CTL mediated lysis). Wildtype-infected cells (X) are additionally destroyed by a CTL clone (or clones), specific for the epitope in which the escape mutation occurs, at rate c. Infected cells produce free virions (at rate h for wildtype-infected cells, and rate h’ for variant-infected cells), which are cleared at rate u. The system was assumed to be at equilibrium prior to the emergence of the variant at time t=0 so the starting populations of uninfected CD4^+^ cells (S), CD4^+^ cells infected with wildtype virus (X) and free wildtype virus (W) are given by the (no variant) equilibrium solution. The starting number of cells infected with the variant (Y) was zero and the starting amount of free variant virus (V) was set to a fraction of the free wildtype virus.

Parameters were randomly sampled from the biological ranges as defined in Table 1 in the main text. Parameter b is the rate of death of infected cells attributable to all the other CTL responses (typically 20) plus CTL independent death (e.g. natural cell death, cytolytic effects of the virus, NK-mediated cell death, etc), and not just CTL-independent death. Parameter c is the rate of death of infected cells attributable to a ***single*** CTL response, namely the CTL response that is escaped (where we define a response as a clone or clones recognising a single epitope), and not the death rate attributable to all CTL.

Two versions of the model were run. Model 1 assumed that the mutant would always be attenuated and as such always has up to 100 fold lower infection (β’) and replication rates (h’) than the wildtype. This was implemented by adjusting the mutant parameter ranges once the wildtype parameters for β and h were chosen. Model 2 assumed that the mutant would very quickly acquire compensatory mutations, which could fully restore the fitness cost of evading CTL recognition. In this model, the mutant parameter ranges were left the same as the wildtype parameter ranges. Parameter sets which did not favour the variant, determined by Routh-Hurwitz local stability conditions for a 5D model [2], were discarded and new parameters were selected.

The model was simulated using *lsoda* in R until 100,000 runs were obtained in which the variant virus reached fixation. A stochastic simulation of this model based on the Crow-Kimura model of mutation and selection gives very good agreement between the estimated time to fix from first detected appearance and the actual time to fix from first detected appearance in the simulation (see p11).

Randomly sampling parameters from wide ranges for both the wildtype and mutant resulted in the generation of mutant virus with a range of different outgrowth rates. The median outgrowth rate of escape variants in HIV infected patients has previously been quantified and observed to lie in the range 0.01-0.04 per day [1]. We therefore calculated the difference in log viral load for runs where the value of k was within this range.

**5D Model – Equal Fitness**

**The 5D model (as described in Methods) was modified so that in the absence of CTL, there was no difference between the fitness of the wildtype and variant. The modified model is given in Equation S1. Parameters and method are otherwise identical to the 5D model.**

(1)

Results

Median log viral load difference of runs (all variants fixed at 100%): 0.024 log

**6D Model – Mass Action Terms (Model A)**

**The 5D model (as described in Methods) was extended to include cytotoxic T-lymphocytes which recognised a single epitope of the wildtype virus as a dynamic 6^th^ population (named ‘E’). This addition also altered the lysis term from ‘cX’ to ‘cEX’. The complete model is given in Equation S2. New and altered parameters are given below, and are otherwise as the 5D model.**

(2)

| **Parameter** | **Min** | **Max** | **Description (units)** | **Ref** |
| --- | --- | --- | --- | --- |
| c | 0.00002 | 0.0002 | Death rate of infected CD4^+^ cells by CTLs recognising a single wildtype epitope before escape occurs (c^-1^ d^-1^) | [1, 3] |
| σ | 0.0005 | 0.0015 | Very small influx of “naïve” CTL cells (c d^-1^) | [4] |
| g | 0.00005 | 0.001 | Proliferation rate of cytotoxic T-lymphocytes in presence of antigen (c^-1^ d^-1^) | [5, 6] |
| δ | 0.01 | 0.1 | Death rate of activated CD8^+^ T-lymphocytes (d^-1^) | [4, 6] |

Units: c: cells mm^-3^; d: day

| **Population** | **Description** | **Starting Population** | |
| --- | --- | --- | --- |
|  |  | **Run 1** | **Run 2** |
| S | Uninfected CD4^+^ cells per mm^3^ | 1000 | S at end of Run 1 |
| X | CD4^+^ cells infected with wildtype virus per mm^3^ | 0 | X at end of Run 1 |
| W | Free wildtype virus per mm^3^ | 10 | W at end of Run 1 |
| Y | CD4^+^ cells infected with variant virus per mm^3^ | 0 | 0 |
| V | Free variant virus per mm^3^ | 0 | 0.1 |
| E | CTL recognising a single epitope on the wildtype per mm^3^ | 0 | E at end of Run 1 |

Method

As with the 5D model, parameters were randomly varied from a uniform distribution. However, starting populations were not calculated from analysis of equilibrium states. Instead, the model was first run with starting populations to establish a wildtype infection (Run 1). The population counts at the end of Run 1 were used to set the starting populations of Run 2. Parameter sets which did not manage to reach a variant fix percentage of at least 50% in Run 2 were rejected.

Results

Median log viral load difference of runs with a minimum fix percentage of 50%: No parameter sets found

Median log viral load difference of runs with a minimum fix percentage of 90%: No parameter sets found

**6D Model – Saturated Terms**

**The mass action 6D model (above) was altered, following Althaus *et al.*** [4]**, to include Michaelis-Menten saturation for the infection, lysis and CTL proliferation terms. The model is given in Equation S3.**

(3)

| **Parameter** | **Min** | **Max** | **Description (units)** | **Ref** |
| --- | --- | --- | --- | --- |
| λ | 10 | 1000 | Influx of new uninfected CD4^+^ cells (c d^-1^) | [4] |
| d | 0.013 | 0.0775 | Natural death rate of uninfected CD4^+^ cells (d^-1^) | [6] |
| β | 10 | 2000 | Infection rate of wildtype virus (c v^-1^ d^-1^) | [4] |
| β' | 10 | 2000 | Infection rate of variant virus (c v^-1^ d^-1^) | [4] |
| b | 0.5 | 1.0 | Death rate of infected CD4^+^ cells (not CTL-mediated) | [7, 8] |
| c | 0.02 | 0.2 | Death rate of infected CD4^+^ cells by CTLs recognising a single wildtype epitope before escape occurs (d^-1^) | [1, 3] |
| h | 0.02 | 10 | Burst rate of CD4^+^ cells infected with wildtype (v c^-1^ d^-1^) | [7, 9] |
| h' | 0.02 | 10 | Burst rate of CD4^+^ cells infected with variant (v c^-1^ d^-1^) | [7, 9] |
| u | 3 | 300 | Clearance rate of free virus (d^-1^) | [10, 11] |
| σ | 0.0005 | 0.0015 | Very small influx of “naïve” CTL cells (c d^-1^) | [4] |
| g | 0.05 | 1.0 | Proliferation rate of cytotoxic T-lymphocytes in presence of antigen (d^-1^) | [5, 6] |
| δ | 0.01 | 0.1 | Death rate of activated CD8^+^ T-lymphocytes (d^-1^) | [4, 6] |
| h_β_ | 10^-6^ |  | Saturation constant for infection rate (not varied) (c) | [4] |
| h_c_ | 100 |  | Saturation constant for lysis rate (not varied) (c) | [4] |
| h_g_ | 10 | 10000 | Saturation constant for proliferation rate (c) | [4] |

Units: c: cells mm^-3^; v: virions mm^-3^; d: day

| **Population** | **Description** | **Starting Population** | |
| --- | --- | --- | --- |
|  |  | **Run 1** | **Run 2** |
| S | Uninfected CD4^+^ cells per mm^3^ | 1000 | S at end of Run 1 |
| X | CD4^+^ cells infected with wildtype virus per mm^3^ | 0 | X at end of Run 1 |
| W | Free wildtype virus per mm^3^ | 10 | W at end of Run 1 |
| Y | CD4^+^ cells infected with variant virus per mm^3^ | 0 | 0 |
| V | Free variant virus per mm^3^ | 0 | 0.1 |
| E | CTL recognising a single epitope on the wildtype per mm^3^ | 0 | E at end of Run 1 |

Method

As detailed for ‘6D Model using mass action dynamics’.

Results

Median log viral load difference of runs with a minimum fix percentage of 50%: 0.032

Median log viral load difference of runs with a minimum fix percentage of 90%: 0.037

**6D Model – Alternative Saturated Terms**

We also investigated the effect changing some of the model terms from mass action kinetics to saturated terms. Parameter ranges are the same as the mass action 6D model (above, designated as Model A below), scaled as required for saturation kinetics. Details of the different models, and the median log viral load difference, are summarised in the table below.

| Model name | Terms | | | | Median log difference in viral load^1^ |
| --- | --- | --- | --- | --- | --- |
|  | Wildtype infection | Variant infection | Lysis | CTL proliferation |  |
| A | $\beta SW$ | $\beta'SW$ | $cEX$ | $gEX$ | NRF (50%) NRF (90%) |
| B | $\beta SW$ | $\beta'SW$ | $\frac{cEX}{h_{c}+E+X}$ | $gEX$ | NRF (50%)  NRF (90%) |
| C | $\frac{\beta SW}{h_{\beta}+S+X+Y}$ |  | $\frac{cEX}{h_{c}+E+X}$ | $\frac{gEX}{h_{g}+E+X}$ | 0.035 (50%)  0.037 (90%) |
| D | $\beta SW$ | $\beta'SW$ | $\frac{cEX}{h_{c}+E+X}$ | $\frac{gEX}{h_{g}+E+X}$ | NRF (50%)  NRF (90%) |
| E | $\beta SW$ | $\beta'SW$ | $cEX$ | $\frac{gEX}{h_{g}+E+X}$ | NRF (50%)  NRF (90%) |

^1^ Figure in brackets indicates the minimum required fix percentage of the variant. NRF indicates no runs found at the given fix percentage, after running 10^6^ iterations.

If a model failed to find any correct runs, it was repeated by setting the variant fitness equal to the wildtype fitness in the absence of CTL, i.e. β’ was set to β and h’ set to h. Results (all at minimum 90% fix) are shown below.

| Model name | Median log difference in viral load (variant fitness equal to wildtype) |
| --- | --- |
|
| A | 0.0290 |
| B | *NRF* |
| C | 0.0173 |
| D | 0.0235 |
| E | 0.0259 |

*NRF: No runs found, after running 10^6^ iterations.*

Supplemental Methods

*Relationship between the outgrowth rate, k, and time to fix*

To estimate the time to fix (*t_fix_*) in terms of the outgrowth rate, k, we use the following expression (Equation S4), which is obtained for the 2D model [1, 3, 12] and provides a good approximation for k in the 5D model (Pearson’s product moment correlation coefficient: p<0.0001, r=0.998):

$\text{proportion of variant (t)}=\frac{1}{1+ge^{-kt}}$ (4)

Where k is the outgrowth rate and g is the size of the wildtype population relative to the variant at time zero (*t_0_*), i.e.

$g=\frac{X(0)}{Y(0)}$ (5)

We use Equation S4 to estimate the time to fix as the time for a variant to grow from 1% at time zero to 99% at time τ, see Equation S6.

$\begin{matrix} \frac{99}{100}= \frac{1}{1+99e^{-k\tau}} \\ -k\tau= \ln\left[\frac{\frac{100}{99}-1}{99}\right] \\ \tau\approx\frac{9}{k} \end{matrix}$ (6)

*Stochastic effects in the 5D mathematical model*

An agent-based simulation of mutation and selection was performed numerically in Maple using a modified Crow-Kimura model [13, 14]. Cells infected with wildtype virus (X) were set at 99% density with a selection coefficient of 1. Wildtype infected cells could mutate at rate u into cells infected with variant virus (Y) with selection coefficient 1+k. k was varied for each run, and the time to first emergence of variant to 1% proportion, and time to variant fixation (at 99% proportion) recorded. The estimated time to fix (9/k, Equation S6) and actual time to fix were in good agreement. The data from each run was also fitted to the variant proportion equation (Equation S4), and a very strong positive correlation between actual k and fitted (stochastic) k was observed. Code is available upon request.

***Stricter definition of escape***

As a more stringent method of estimating NEE, we used the single additional criterion of a reduction in the “epitope score” score of ≥50% of the mutant when compared with the cohort consensus. The “epitope score” is derived using predictive software NetMHC v3.0 [15, 16] and NetCTL [17] and is based on epitope-allele binding affinity, TAP transport and proteasomal cleavage scores. **Predictive software has been shown to achieve a high level of accuracy** [18-22] **and the correlation between predicted affinity and experimentally determined affinity is as good as the correlation between experimental measurements from different laboratories** [23]**. Epitope prediction software is increasingly being used in the field of HIV-1 immunology** [24-26] **and more generally in CD8+ T cell epitope discovery** [27, 28]**.**

***Increasing the number of HLA-epitopes for gag***

This list is taken from Rousseau *et al.* 2008 [29], and comprises of 39 “escape” codons from *gag* in the Durban cohort, where escape is interpreted as pressure to change away from the wildtype codon in the presence of the restricting HLA type. This list was constructed using a phylogenetic corrected method. Codons associated with the same HLA allele and within 9 amino acids of another codon were deemed to lie within the same epitope, and so not counted separately.

***Removing frequent or protective alleles***

**If a frequent allele was driving wildtype sequence to the escape mutation, then the NEE metric would identify wildtype sequence (i.e. susceptible to the CTL response) as escape events. Recent work** [30] **suggests that this may be truer for protective alleles rather than frequent alleles. We define a frequent allele as** occurring in more than 6% of the cohort. An allele was defined as ‘protective’ if the median viral load of all individuals possessing that allele was lower than the median viral load of the cohort, see Figure 3 of Kiepiela *et al.* [31]. Individuals with protective or frequent alleles were removed from the cohort and the analysis repeated.

***Escape events in which gene drive association between escape and log viral load?***

The original cohort was bootstrapped 10,000 times, by sampling individuals with replacement.

For each bootstrap run, if escape events over all genes in the bootstrapped cohort was a significant predictor of log viral load, independently of the number of synonymous changes over all genes, then escape events were split by gene to determine which gene was the significant predictor of log viral load, again with the number of synonymous changes as a co-predictor. There are three cases:

1. No single gene is a significant independent predictor of log viral load: The bootstrap run is marked as “No gene is a significant predictor of vl”.
2. One gene is a significant independent predictor of log viral load: The bootstrap run is marked as being driven by the single gene.
3. Multiple genes are significant predictors of log viral load: All significant genes are tested in multiple linear regression to determine whether they are independent predictors of log viral load. There are two cases:
   1. No gene retains significance independently: The bootstrap run is marked as being driven by the gene with the lowest p-value.
   2. One or more genes retain significance: The bootstrap run is marked as being driven by all genes retaining significance independently.

Once the driving gene(s) have been identified for each bootstrap run where escape events in all genes were a significant independent predictor of log viral load, sets of genes contributing to less than 1% of all significant runs were grouped together as “Minor effects”. These sets of genes did not further contribute to the analysis.

For each run where the number of escape mutations in all genes was a statistically significant predictor of log viral load, we identified the gene responsible for this association. The breakdown by percentage for each gene is shown in Table S5. Note that these do not sum to 100%, as escape events in multiple genes can be independent significant predictors of log viral load for a single bootstrap run.

*Variance in viral load due to random fluctuations*

We wanted to know how much the viral load in an individual varied over time due to random fluctuations. To do this, we measured the variance in viral load in a longitudinal B-clade HIV-infected cohort from Switzerland, detailed in Fryer *et al.* 2009 [32]. We calculated the variance of the log viral load per person of viral loads taken 3 months after the last treatment (from week 48 onwards). We find that the median standard deviation in log viral load is 0.32 (95% confidence interval: 0.27-0.35; range: 0.049-0.95; n=52).

*Fraction of viral load variation explained by HLA class I genotype*

We quantified the proportion of viral load variation that could be explained by HLA class I genotype using four different sources:

1. **Nelson: 3-5%**

Nelson *et al.* [33] described the Explainable Fraction (EF) metric and calculated the contribution of different genetic factors affecting progression to AIDS. By summing the protective factors (HLA-B*27 and HLA-B*57) and susceptible factors (1 x HLA class I homozygosity, 2-3 x HLA class I homozygosity and HLA-B*35Px), we can quantify the effect of the 5 most significant HLA class I factors on three different AIDS endpoints:

| **AIDS Endpoint** | **Explainable Fraction** |
| --- | --- |
| CD4^+^ count of less than 200 cells/ml | 3.1 |
| CDC 1993 AIDS definition | 3.6 |
| CDC 1987 AIDS definition | 5.1 |

1. **Pereyra: 3%**

We applied the EF metric of Nelson *et al.* [33] to the data reported by Pereyra *et al.* [34]. First, we created a contingency table of casual factors and disease categories. Class I HLA genotypes were categorised into ‘protective’ (including B*57, B*27, B*5801, B*1503, B*13 and B*51) and ‘not protective’ casual factors. Patient long-term viral load was used as the disease categories (elite controller: <50 RNA copies/ml; viremic controller: 50-2,000 RNA copies/ml; chronic progressor: >10,000 copies/ml).

Data was reported as the frequency of each disease class having at least one protective allele, resulting in the following contingency table:

|  | Elite controller | Viremic controller | Chronic progressor |
| --- | --- | --- | --- |
| Protective HLA | 0.68 | 0.6 | 0.37 |
| No protective HLA | 0.32 | 0.4 | 0.63 |
| **Total:** | **1** | **1** | **1** |

The frequencies were then normalised across the columns according to frequency in the HIV-infected populace (elite: 0.003333 [35]; viremic: 0.1 [36]; chronic: 0.8967):

|  | Elite controller | Viremic controller | Chronic progressor | **Row marginals** |
| --- | --- | --- | --- | --- |
| Protective HLA | 0.002267 | 0.06 | 0.3318 | **0.3940** |
| No protective HLA | 0.001067 | 0.04 | 0.5649 | **0.6060** |
| **Column marginals** | **0.003333** | **0.1** | **0.8967** | **1** |

We then calculated the mutual information, I(1,2):

| **I(1,2)** | Elite controller | Viremic controller | Chronic progressor |
| --- | --- | --- | --- |
| Protective HLA | 0.001237 | 0.02523 | -0.02088 |
| No protective HLA | -0.0006811 | -0.01661 | 0.02197 |

And the maximised mutual information metric, I_max_(1,2) ,for each disease category:

|  | Elite controller | Viremic controller | Chronic progressor |
| --- | --- | --- | --- |
| I_max_(1,2) | -0.01901 | -0.2303 | -0.09780 |

The EF for the HLA casual factors on disease status is then the sum of I(1,2), divided by the sum of I_max_(1,2). This results in an EF of 0.02957, or 3%.

1. **Emu: 9%**

We also applied the EF metric of Nelson *et al.* [33] to the data reported by Emu *et al.* [37]. First, we created a contingency table of casual factors and disease categories. Class I HLA genotypes were categorised into ‘protective’ (including B*13, B*27, B*57, B*58, B*81) and ‘not protective’ casual factors. Patient long-term viral load was used as the disease categories (elite controller: <75 RNA copies mL^-1^; viremic controller: <2,000 RNA copies mL^-1^; chronic progressor: >10,000 copies mL^-1^).

Data was reported as the frequency of each disease class having at least one protective allele, resulting in the following contingency table:

|  | Elite controller | Viremic controller | Chronic progressor |
| --- | --- | --- | --- |
| Protective HLA | 0.60 | 0.52 | 0.16 |
| No protective HLA | 0.40 | 0.48 | 0.84 |
| **Total:** | **1** | **1** | **1** |

The frequencies were then normalised across the columns according to frequency in the HIV-infected populace (frequencies as for Pereyra):

|  | Elite controller | Viremic controller | Chronic progressor | **Row marginals** |
| --- | --- | --- | --- | --- |
| Protective HLA | 0.002000 | 0.052 | 0.1435 | **0.1975** |
| No protective HLA | 0.001333 | 0.048 | 0.7532 | **0.8025** |
| **Column marginals** | **0.003333** | **0.1** | **0.8967** | **1** |

We then calculated the mutual information, I(1,2):

| **I(1,2)** | Elite controller | Viremic controller | Chronic progressor |
| --- | --- | --- | --- |
| Protective HLA | 0.002223 | 0.05035 | -0.03018 |
| No protective HLA | -0.0009284 | -0.02467 | 0.03437 |

The maximised mutual information metric, I_max_(1,2), is the same as for Pereyra. The EF for the HLA casual factors on disease status is then the sum of I(1,2), divided by the sum of I_max_(1,2). This results in an EF of 0.08977, or 9%.

1. **Fellay: 6-15%**

Fellay *et al.* [38] used a whole genome analysis to identify host polymorphisms that explain between-individual variation in viral load. In a sub-cohort of 187 individuals with four digit HLA class I typing results, they estimated that HLA class I alleles together explained between 6% and 15% of the observed viral load variation (Fellay, *personal communication*).

We estimate proportion of viral load variation that could be explained by HLA class I genotype to be 3-15%, obtained from the range of EFs from the above sources.

Supplemental References

1. Asquith B, Edwards CTT, Lipsitch M, McLean AR. (2006) Inefficient cytotoxic T lymphocyte-mediated killing of HIV-1-infected cells in vivo. PLoS Biol 4: e90.

2. May RM. (1974) Stability and complexity in model ecosystems. Princeton University Press.

3. Mandl JN, Regoes RR, Garber DA, Feinberg MB. (2007) Estimating the effectiveness of simian immunodeficiency virus-specific CD8+ T cells from the dynamics of viral immune escape. J Virol 81: 11982-91.

4. Althaus CL, De Boer RJ. (2008) Dynamics of immune escape during HIV/SIV infection. PLoS Comput. Biol 4: e1000103.

5. Davenport MP, Ribeiro RM, Perelson AS. (2004) Kinetics of virus-specific CD8+ T cells and the control of human immunodeficiency virus infection. J Virol 78: 10096―10103.

6. Macallan DC, Asquith B, Irvine AJ, Wallace DL, Worth A, et al. (2003) Measurement and modeling of human T cell kinetics. Eur J Immunol 33: 2316-26.

7. Müller V, Marée AF, De Boer RJ. (2001) Small variations in multiple parameters account for wide variations in HIV-1 set-points: a novel modelling approach. Proc Biol Sci 268: 235-42.

8. Markowitz M, Louie M, Hurley A, Sun E, Di Mascio M, et al. (2003) A novel antiviral intervention results in more accurate assessment of human immunodeficiency virus type 1 replication dynamics and T-cell decay in vivo. J Virol 77: 5037-8.

9. Haase AT. (1999) Population biology of HIV-1 infection: viral and CD4+ T cell demographics and dynamics in lymphatic tissues. Annu Rev Immunol 17: 625-56.

10. Perelson AS, Neumann AU, Markowitz M, Leonard JM, Ho DD. (1996) HIV-1 dynamics in vivo: virion clearance rate, infected cell life-span, and viral generation time. Science 271: 1582-6.

11. Zhang ZQ, Notermans DW, Sedgewick G, Cavert W, Wietgrefe S, et al. (1998) Kinetics of CD4+ T cell repopulation of lymphoid tissues after treatment of HIV-1 infection. Proc Natl Acad Sci U S A 95: 1154-9.

12. Ganusov VV, Boer RJD. (2006) Estimating Costs and Benefits of CTL Escape Mutations in SIV/HIV Infection. PLoS Comput Biol 2: e24.

13. Crow J, Kimura M. (1970) An introduction to population genetics theory. Harper & Row.

14. Asquith B. (2008) The evolutionary selective advantage of HIV-1 escape variants and the contribution of escape to the HLA-associated risk of AIDS progression. PLoS ONE 3: e3486.

15. Buus S, Lauemøller SL, Worning P, Kesmir C, Frimurer T, et al. (2003) Sensitive quantitative predictions of peptide-MHC binding by a 'Query by Committee' artificial neural network approach. Tissue Antigens 62: 378―384.

16. Nielsen M, Lundegaard C, Worning P, Lauemøller SL, Lamberth K, et al. (2003) Reliable prediction of T-cell epitopes using neural networks with novel sequence representations. Protein Sci 12: 1007-17.

17. Larsen MV, Lundegaard C, Lamberth K, Buus S, Brunak S, et al. (2005) An integrative approach to CTL epitope prediction: a combined algorithm integrating MHC class I binding, TAP transport efficiency, and proteasomal cleavage predictions. Eur J Immunol 35: 2295―2303.

18. Schellens IMM, Keşmir C, Miedema F, van Baarle D, Borghans JAM. (2008) An unanticipated lack of consensus cytotoxic T lymphocyte epitopes in HIV-1 databases: the contribution of prediction programs. AIDS 22: 33-7.

19. Fortier M, Caron E, Hardy M, Voisin G, Lemieux S, et al. (2008) The MHC class I peptide repertoire is molded by the transcriptome. J Exp Med 205: 595-610.

20. Pérez CL, Larsen MV, Gustafsson R, Norström MM, Atlas A, et al. (2008) Broadly immunogenic HLA class I supertype-restricted elite CTL epitopes recognized in a diverse population infected with different HIV-1 subtypes. J Immunol 180: 5092-100.

21. Larsen MV, Lundegaard C, Lamberth K, Buus S, Lund O, et al. (2007) Large-scale validation of methods for cytotoxic T-lymphocyte epitope prediction. BMC Bioinformatics 8: 424.

22. MacNamara A, Kadolsky U, Bangham CRM, Asquith B. (2009) T-cell epitope prediction: rescaling can mask biological variation between MHC molecules. PLoS Comput. Biol 5: e1000327.

23. Peters B, Bui H, Frankild S, Nielson M, Lundegaard C, et al. (2006) A community resource benchmarking predictions of peptide binding to MHC-I molecules. PLoS Comput Biol 2: e65.

24. Brumme ZL, Brumme CJ, Heckerman D, Korber BT, Daniels M, et al. (2007) Evidence of differential HLA class I-mediated viral evolution in functional and accessory/regulatory genes of HIV-1. PLoS Pathog 3: e94.

25. Borghans JAM, Mølgaard A, Boer RJD, Keşmir C. (2007) HLA Alleles Associated with Slow Progression to AIDS Truly Prefer to Present HIV-1 p24. PLoS ONE 2: e920.

26. Schmid B, Keşmir C, de Boer RJ. (2008) The specificity and polymorphism of the MHC class I prevents the global adaptation of HIV-1 to the monomorphic proteasome and TAP. PLoS ONE 3: e3525.

27. Moutaftsi M, Peters B, Pasquetto V, Tscharke DC, Sidney J, et al. (2006) A consensus epitope prediction approach identifies the breadth of murine T(CD8+)-cell responses to vaccinia virus. Nat Biotechnol 24: 817-9.

28. Sundar K, Boesen A, Coico R. (2007) Computational prediction and identification of HLA-A2.1-specific Ebola virus CTL epitopes. Virology 360: 257-63.

29. Rousseau CM, Daniels MG, Carlson JM, Kadie C, Crawford H, et al. (2008) HLA class I-driven evolution of human immunodeficiency virus type 1 subtype c proteome: immune escape and viral load. J Virol 82: 6434-46.

30. Kawashima Y, Pfafferott K, Frater J, Matthews P, Payne R, et al. (2009) Adaptation of HIV-1 to human leukocyte antigen class I. Nature 458: 641-645.

31. Kiepiela P, Leslie AJ, Honeyborne I, Ramduth D, Thobakgale C, et al. (2004) Dominant influence of HLA-B in mediating the potential co-evolution of HIV and HLA. Nature 432: 769-775.

32. Fryer HR, Scherer A, Oxenius A, Phillips R, McLean AR. (2009) No evidence for competition between cytotoxic T-lymphocyte responses in HIV-1 infection. Proceedings of the Royal Society B: Biological Sciences 276: 4389-4397.

33. Nelson GW, O'Brien SJ. (2006) Using mutual information to measure the impact of multiple genetic factors on AIDS. J Acquir Immune Defic Syndr 42: 347-54.

34. Pereyra F, Addo MM, Kaufmann DE, Liu Y, Miura T, et al. (2008) Genetic and immunologic heterogeneity among persons who control HIV infection in the absence of therapy. J Infect Dis 197: 563-71.

35. Walker BD. Elite control of HIV Infection: implications for vaccines and treatment. Top HIV Med 15: 134-6.

36. Fraser C, Hollingsworth TD, Chapman R, Wolf FD, Hanage WP. (2007) From the Cover: Variation in HIV-1 set-point viral load: Epidemiological analysis and an evolutionary hypothesis. Proc Natl Acad Sci U S A 104: 17441―17446.

37. Emu B, Sinclair E, Hatano H, Ferre A, Shacklett B, et al. (2008) HLA class I-restricted T-cell responses may contribute to the control of human immunodeficiency virus infection, but such responses are not always necessary for long-term virus control. J Virol 82: 5398-407.

38. Fellay J, Shianna KV, Ge D, Colombo S, Ledergerber B, et al. (2007) A whole-genome association study of major determinants for host control of HIV-1. Science 317: 944-7.
